# From Single Ligand–Receptor Bond Strength to Collective Avidity: Mechanics-Guided Superselective Nanoparticle Adhesion to Biological Membranes

**DOI:** 10.1021/acs.langmuir.5c03244

**Published:** 2025-12-16

**Authors:** Morteza Hamzeh, Saba Mirahsani, Fatemeh Ahmadpoor, Samaneh Farokhirad

**Affiliations:** Department of Mechanical and Industrial Engineering, 5965 New Jersey Institute of Technology, Newark, New Jersey 07114, United States

## Abstract

Multivalent adhesion between ligand-coated nanoparticles (NPs) and cell membrane receptors is central to targeted nanomedicine, yet how NP mechanics tune the classic affinity-selectivity trade-off remains unclear. Here we combine Monte Carlo simulations with thermodynamic analysis to probe the binding free-energy landscape of rigid, semirigid, and deformable NPs interacting with target receptors. By sweeping membrane tension, receptor density, and ligand–receptor affinities (spanning the full weak-to-strong regime), we uncover a mechanics-governed switch in optimal design. The entropy-enthalpy compensation reveals that deformable NPs dominate at intermediate affinities, exploiting shape adaptability to recruit nearly all available receptors even at low expression levels, albeit at significant entropic cost. The semirigid NPs, in turn, require the strongest affinity to offset configurational penalties and maximize avidity, while rigid NPs never engage more than ∼10% of their ligand capacity. The interactions under weak individual bonds fail to nucleate adhesion under any mechanical or biochemical condition tested. Additionally, increasing membrane tension selectively suppresses multivalency of binding for rigid and semirigid NPs but leaves deformable NPs largely unaffected. The results collapse onto mechanics-affinity phase diagrams that can predict design windows where (super) selective adhesion emerges from the interplay of NP stiffness, membrane deformation, bond strength, and receptor density. These insights provide quantitative guidelines for engineering deformable, affinity-tuned nanocarriers capable of high selectivity under physiologically relevant mechanical and biochemical heterogeneity.

## Graphical abstract

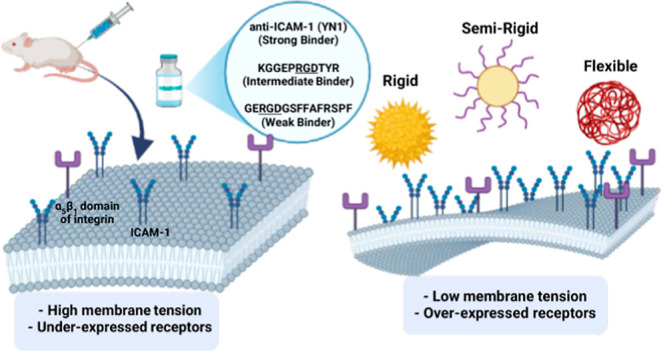

## Introduction

Multivalent interactions, where multiple ligands and receptors cooperatively form strong and highly specific bonds, are prevalent throughout biological systems. Examples range from intrinsically disordered protein–protein interactions^1–4^ and ubiquitylation processes^5^ to highly selective antibody–antigen interaction^6^ and virus-host binding events^7,8^ such as bacteriophage adhesion to bacterial surfaces. Individually, these ligand–receptor interactions are typically weak and reversible, yet collectively they establish strong, selective, and biologically critical binding events. This cooperative binding capability forms the foundation for numerous biological phenomena,^9–15^ including receptor trafficking, signal transduction, and cell adhesion, thereby governing cellular responses to external stimuli and influencing various cell fates. Harnessing the specificity and selectivity inherent to multivalent interactions has drove advancements in biotechnology and nanomedicine, particularly in targeted drug delivery, diagnostics, and biosensing applications.^16–23^ Modern experimental platforms, including polymers,^24,25^ viruses,^7^ nanoparticles (NPs),^26–28^ and microparticles,^29,30^ exploit multivalency to achieve selective targeting of cell surfaces based on membrane receptor signatures that are characteristic of specific cell types.

A parallel, often underappreciated, design axis is the monovalent binding strength of each ligand–receptor pair, which can be classified as strong, intermediate, or weak binding systems. These binding affinities not only set the baseline for cellular adhesion, signal transduction, and immune recognition in vivo^31^ but also shift in response to local factors (i.e., pH, temperature, ionic milieu),^32–34^ thereby tuning multivalent interactions. In addition, the density of surface receptors, known as expression level, on target cells can vary by orders of magnitude between healthy and diseased cells^35,36^ and directly control the threshold of NP binding strength, enabling receptor density-dependent selectivity.

Significant advances have been achieved in understanding the high selectivity of biological systems upon multivalent receptor–ligand specificity, particularly emphasizing the interplay of binding energies, ligand distributions, and entropy-enthalpy compensation. Martinez-Veracoechea and Frenkel laid the theoretical groundwork, introducing statistical mechanical frameworks that highlight the concept of superselectivity, a nonlinear increase in binding probability under conditions of weak individual interactions coupled with high multivalency.^37^ Computational models by Curk et al.^38^ and Dubacheva et al.^24^ further refined these concepts, elucidating the roles of the type and precise distribution of ligand–receptor interactions. Experimentally, these theoretical insights have been validated and expanded upon through various model systems. Scheepers et al. experimentally confirmed predictions using DNA-coated colloidal NPs, with weaker ligand–receptor affinities exhibiting enhanced selectivity.^30^ Albertazzi et al.^39^ and Dubacheva et al.^24,25^ provided additional experimental demonstrations of superselectivity using supramolecular polymers and polymer systems, reinforcing the theoretical predictions. Further insights from Linne et al., using DNA nanostars, elucidated that cooperative ligand–ligand interactions and specific valency levels play key roles in achieving superselective binding.^40^ McKenzie et al. employed molecular dynamics simulations to investigate how ligand composition, and receptor–ligand binding strength influence multivalent adhesion, with a particular focus on the balance between enthalpic gains and entropic losses.^41^ They demonstrated that strong-binding ligands require favorable mechanical conditions, such as flexible bond stiffness, to maintain binding. Their findings reveal that tuning ligand composition and NP shape can shift this balance to enhance multivalent interactions. Xia et al. expanded these insights through Monte Carlo simulations and analytical mean-field theory, identifying conditions where intermediate binding strengths achieve higher selectivity than traditionally favored weak interactions, especially in hyperuniform receptor distributions.^42^

Despite these advances, the role of NP deformability in conjunction with membrane mechanics in determining multivalent NP-cell interactions remain insufficiently explored. Relatively few experimental and modeling studies have examined how cell adhesion and delivery of NPs by multivalent system depends on NP deformability.^43–48^ Additionally, the mechanical properties of cell membranes influence these multivalent interactions. Different cell types exhibit unique membrane mechanical properties, such as membrane tension (or excess area), defining distinct mechanotypes, which could be leveraged to optimize multivalent NP targeting, enhancing specificity or selectively avoiding undesirable interactions. Experimental studies frequently utilize supported lipid bilayers to mimic the fluid and dynamic properties of biological membranes, revealing the essential role receptor mobility plays in facilitating multivalent ligand–receptor engagements.^40^ Conversely, computational/theoretical studies have often simplified the cells as rigid substrates, though some advanced frameworks have considered cell flexibility to enhance biological realism.^41^ As such, focusing on the potential benefits of deformable NPs by controlling and tuning the NP stiffness for a desired selectivity and specificity of binding to target cells with unique mechanotypes is essential and can offer a lot of exciting opportunities in design and performance in vivo.

In this article, we employ a statistical-mechanics-based computational framework to link NP deformability, membrane mechanics, and receptor expression levels, enabling us to predict multivalent interactions and the binding of ligands immobilized on NP surface to receptors diffusing on undulating cell-membrane models across a wide range of ligand–receptor affinities. We have chosen endothelial cells as our membrane models and functionalized them with target receptors.

A main barrier to the rational design of NPs, functionalized by targeting molecules or drugs, for in vivo use is that the mapping between the displayed efficacy of binding and design/tunable parameters is nonlinear, and in many cases show the opposite behavior to conventional wisdom owing to large contributions from entropic forces. Employing our recently proposed thermodynamic methods,^28^ we address these dependencies through investigations of the interplay between the enthalpic gain due to the ligand–receptor binding and entropic cost associated with conformational degrees of freedom. The degrees of freedom include receptor translation and flexure, NP configuration, membrane undulation, and NP translation and rotation. By elucidating these critical interactions, we aim to provide guiding principles for the rational design of deformable, affinity-tuned nanocarriers with enhanced specificity and efficacy in vivo.

## Materials and Methods

### Description of the Model

Our previously validated multiscale computational framework^28^ was employed to examine the binding interactions between ligand-coated NPs and receptors diffusing on cell membranes (details provided in the Supporting Information). Briefly, the model integrates equilibrium statistical mechanics, continuum representations of cell membranes, and coarse-grained molecular descriptions for ligands, receptors, and deformable NPs. Receptors are represented as flexible rods, each characterized by a flexural stiffness activated upon ligand binding. The cell membrane is modeled as a triangulated elastic surface governed by the Helfrich Hamiltonian,^49^ capturing bending rigidity and membrane tension effects. Three NP types are considered: rigid (spherical, ligand-coated), semirigid (spherical core with polymeric tether-attached ligands), and deformable (polymeric core–shell NPs, capable of internal structural rearrangements). The deformable NPs are modeled using a bead–spring representation, allowing tunable mechanical stiffness through varying internal cross-linking density. Ligand–receptor interactions are quantified using the Bell bond potential, accounting for bond stiffness and equilibrium binding distances.

To compute the binding free energy (Δ*F*), we decompose it into enthalpic and entropic contributions: Δ*F* = Δ*H*–*T*Δ*S*. The enthalpy of binding (Δ*H*) is computed as the ensemble average over system conformations sampled using Metropolis Monte Carlo (MC) simulations, which incorporate membrane deformation, receptor flexure, ligand–receptor binding, and (for deformable NPs) internal structural interactions. The entropy change (Δ*S*) is calculated from simulation trajectories and includes three configurational terms: NP entropy from quasiharmonic analysis of bead fluctuations, membrane entropy via vertex fluctuation analysis, and receptor translational entropy using the two-dimensional Sackur–Tetrode equation. These components are evaluated separately for bound and unbound ensembles under identical protocols. A detailed description of the thermodynamic calculations is provided in the Supporting Information.

### Parameter Estimation for MC Simulation

#### Nanoparticles

Four distinct NP models are considered to explore the role of mechanical properties and surface structure in their multivalent binding interactions with membranes. The first two are polymeric core–shell NPs, consisting of lysozyme-core and dextran-shell. Following an experimental protocol,^50^ the stiffness of these deformable NPs is tuned by physically cross-linking the polymer chains to create a low-stiffness (LSt) core–shell NP with an elasticity modulus of 0.43 kPa, which is highly deformable due to the absence of cross-linking, and a high-stiffness (HSt) core–shell NP with an elasticity modulus of 15.02 kPa, which has a high degree of internal cross-linking that limits deformability. The other two NP models are a rigid NP (RG) represented as a solid, nondeformable sphere, and a semirigid NP (SRG), consisting of a rigid core with flexible polymeric tethers anchored to its surface. An illustrative schematic depicting the interaction of these distinct NP models with the cell membrane is presented in Figure 1 (see also the Supporting Information for the details of the coarse-grained models for NPs).

**Figure 1:**
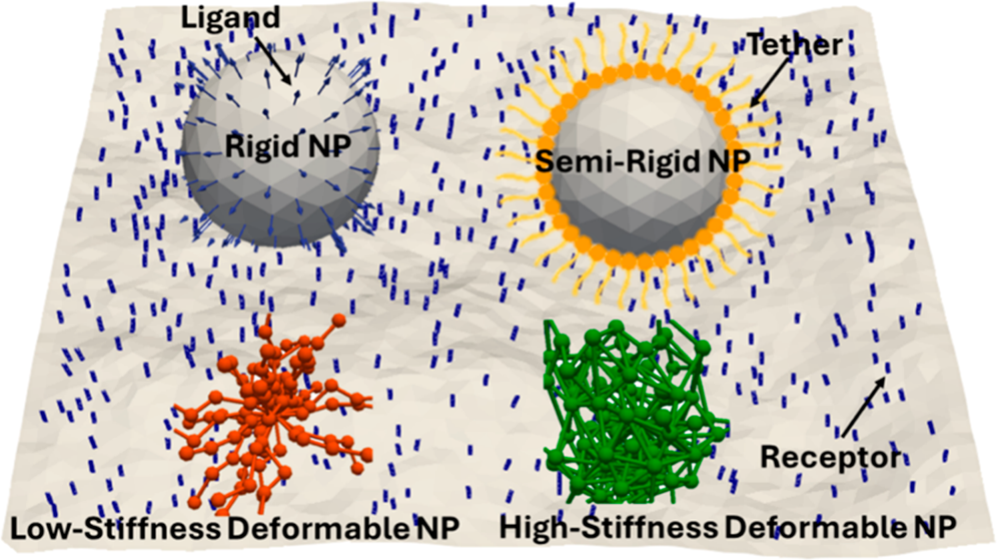
Schematic illustration of the computational setup modeling for four distinct ligand-coated NPs (rigid, semirigid, low-stiffness core–shell, and high-stiffness core–shell NPs) interacting with an undulating cell membrane functionalized with specific receptors, highlighting ligand–receptor multivalent binding dynamics.

### Membrane

The equilibrium conformations of the cell membrane are governed by two key physicochemical parameters in our model: the bending rigidity *k*, and the excess area *A*_ex_. We set the bending rigidity to *k* = 20*k*_*B*_ *T*.^51^ The dimensionless quantity *A*_ex_, defined as *A*_ex_ = (*A*–*A*_*p*_)/*A*, represents the additional surface area available relative to the projected area and thus serves as a measure of membrane tension. Here, *A* is the total curvilinear area, and *A*_*p*_ is the projected area of the equilibrated membrane patch. To obtain the membrane configurations for different *A*_ex_, we keep *A*_*p*_ fixed and vary *A*. The range of *A*_ex_ explored here is 6% to 20%, a span observed in most endothelial cells.^52^

### Receptor

While all our studies were performed with a constant ligand density of 14%, we chose five values for receptor densities *N*_*r*_ = 200, 500, 1000, 2000, and 4000 per μm^2^ of membrane area, as reported in experiments.^53–56^

We note that, to reduce computational cost, we simulated a 500 × 500nm^2^ periodic membrane patch (0.25μm^2^), corresponding to one-quarter of a 1μm^2^ reference membrane area.^53–56^ The use of periodic boundary conditions in the membrane plane ensures the membrane behaves as a seamlessly continuous surface. The NP is modeled in full and placed within the simulation domain such that it can interact with the membrane and receptors naturally across the entire domain. Receptors are counted and distributed according to the full 1μm^2^ scale, but only a quarter of them are present in the 0.25 μm^2^ periodic box during simulation (i.e., *N*_*r*_ = 50, 125, 250, 500, and 1000 per 0.25μm^2^).

### Receptor–ligand Pairs

To analyze the effect of receptor–ligand binding strengths, we focused on three specific receptor–ligand pairs categorized as strong, intermediate, and weak binders. These categories are defined based on their specific interaction free energy. It is also important to note that the receptor–ligand binding stiffness, *k*_*b*_, varies across these categories, reflecting the mechanical response of the receptor–ligand pairs under deformation. Table 1 summarizes the three receptor–ligand pairs used in our study, detailing their respective interaction energies and mechanical stiffness. These pairs are chosen to model biologically relevant binding scenarios. The strong binder system represents the interaction between intracellular adhesion molecules (ICAM1) and the YN1 ligand, a well-studied high-affinity pair involved in immune cell adhesion. The intermediate binder and weak binder systems correspond to interactions between the α_5_β_1_ integrin domain and peptides that mimic fibronectin and collagen, respectively, key components of the extracellular matrix.^57–60^ These interaction models are relevant not only for the rational design of functionalized NPs for targeted delivery, but also for studying cell adhesion mechanics, which are fundamental to many physiological and pathological processes.

**Table 1:** Three Receptor–Ligand Binder Systems

Binder strength	Receptor	Ligand sequence	Binding free energy Δ ***G***_0_ (***k***_***B***_ ***T***)	Binding stiffness ***k***_***b***_ (***N***/***m***)
**Strong**	ICAM-1	anti-ICAM1 (YN1)	–19.1 ^61^	1 ^41,61^
**Intermediate**	α_5_β_1_ domain of integrin	KGGEPRGDTYR	–11.45 ^57^	0.38 ^41^
**Weak**	α_5_β_1_ domain of integrin	GERGDGSFFAFRSPF	–1.76 ^58^	0.56 ^41^

### Numerical Setup

The computational setup consists of a simulation domain measuring 500 × 500 × 620 nm^3^, with periodic boundary conditions applied along the membrane plane. To ensure proper equilibration, the membrane and NPs are first equilibrated separately for 5 × 10^7^ MC steps. Afterward, the NP is positioned near the membrane surface, and the system undergoes relaxation over 5 × 10^8^ MC steps before data collection begins. Each ensemble is 1 × 10^9^ steps of sampling, and for each set of conditions, we generate four independent simulation trajectories. The error bars in the free-energy calculations denote the SD over the four ensembles. We consider the thermodynamic ensemble characterized by constant temperature (*T*), projected membrane area (*A*_*p*_), and membrane tension (σ). This choice of ensemble ensures that the thermodynamic properties of the membrane, particularly its deformation and response to ligand–receptor interactions, are captured in a physically consistent manner. The computational runtime for a typical trajectory is approximately 10 h on a 4.8 GHz processor for the rigid NP case, and up to 2 weeks for the semirigid and deformable NP cases. The detailed parameter sets used in our model are obtained from various sources in the literature and are presented in Table 2.

**Table 2:** Details of the System Parameters for Binding of Functionalized NPs to Cell Surface Receptors

Property	Symbol	Value
Membrane surface area	*L* ^2^	0.25 μm^2^
Mass of each membrane vertex	*m*_*v*_	5.5 × 10^–19^g
Number of links	*N*_links_	7699
Number of membrane vertices	*N*_*v*_	2702
Receptor length	*L*_ant_	19 nm
Number of receptors per 0.25 ***μm*** ^2^	*N*_*r*_	50–1000
Receptor flexural rigidity	*k*_*f*_	7000 pN.nm^2^
Number of ligands per NP	*N*_*l*_	162
Ligand length	*L*_*l*_	15 nm
Bending rigidity	κ	20*k*_*B*_ *T*
Core–Shell deformable NP	**Symbol**	**Value**
Bead radius	*a*_bead_	6.8 nm
Bead mass	*m*_*b*_	1.44 × 10^–19^g
Number of arms attached to the core	*f*	25
Number of beads in each arm	*N*_*b*_	4
Molecular weight of Dextran monomer	*M*_monomer_	160 Da
Molecular weight of Dextran polymer	*M*_polymer_	71 kDa
Size of each Kuhn segment	*b*_*k*_	0.44 nm
Size of monomer	*b*	1.5 nm
Number of monomers per bead	*N*	108
Number of Kuhn segments per bead	*N*_*k*_	246
Stiffness of spring between beads	*k*_*s*_	1.74 × 10^–4^J/m^2^
Semi-rrigid NP	**Symbol**	**Value**
Radius	*R*_SRG_	50 nm
Stiffness	*k*_SRG_	3.3 × 10^–4^J/m^2^
Rigid NP	**Symbol**	**Value**
Radius	*R*_RG_	50 nm
Stiffness	*k*_RG_	1 J/m^2^

### Analysis

To characterize the binding interactions of ligand-functionalized NPs with cell membrane receptors, we conducted a comprehensive thermodynamic analysis. This analysis involved two primary approaches: (a) statistical examination of the multivalent interactions, by calculating the probability distributions and average number of simultaneous ligand–receptor bonds; and (b) direct computation of the adhesion free energy landscape. This allowed us to assess the impact of NP rigidity, receptor density, membrane mechanics, and receptor–ligand binder conditions on multivalent binding behavior and overall binding efficacy.

## Results

### Model Validation

Our multiscale MC and/or coarse-grained molecular dynamics (CGMD) framework has been validated previously for deformable and SRG NPs interacting with thermally fluctuating membranes,^28,46,62^ a summary of relevant studies that include experimental validation is provided in Table S1 (Supporting Information). To complement this and provide validation of our framework for RG NP case, we performed a comparison for RG NPs interacting with the membrane formulation of McKenzie et al.^41^ Their model represents membrane compliance by a mean-field harmonic spring with stiffness *K*_*m*_, which combines in series with the receptor–ligand bond stiffness *k*_*b*_ to define an effective stiffness ${k_{eff}=k}_{b}k_{m}/\left(k_{b}+k_{m}\right)$ . By adopting the same membrane model, we recovered the same multivalency trends for RG NPs over the same range of *k*_eff_ ∼ 10^–3^–1*N*/*m*, as shown in Figure S1. We note that subsequent Results sections use our default model, which couples a fluctuating membrane to NPs of varying rigidity (RG, SRG, and deformable), and reports side-by-side comparisons across these three types of NPs.

### Interplay of NP Rigidity and Receptor Density on NP Avidity to Membrane at ***A***_**ex**_ = 20%

We begin our analysis by exploring the avidity profile of multivalent NPs interacting with cell membranes under strong, intermediate, and weak binders of receptor–ligand affinity systems. To characterize the equilibrium states and dynamic fluctuations of both NPs and cell membranes, By systematically varying receptor density (i.e., *N*_*r*_ = 200, 500, 1000, 2000, and 4000 per μm^2^), mimicking low to high expression levels, we aim to elucidate how NP rigidity and binder strength influence the multivalency distribution and, ultimately, NP avidity. This analysis was conducted under a high membrane excess area of *A*_ex_ = 20% and a constant ligand density of 14% (corresponding to 162 ligands per NP).

### Multivalency of Binding for Strong Binder System

Figure 2 presents the probability distribution of the number of simultaneous bonds between ligand–receptor pairs, *P*(*m*), under strong binding conditions for RG, SRG, and deformable NPs at *N*_*r*_ = 250, 500, and 1000. Additional cases for *N*_*r*_ = 50 and 125 are shown in Figure S2 (Supporting Information). For RG NPs (Figure 2A), the multivalency distributions evolve gradually with increasing receptor density but remain constrained by the NP’s lack of deformability. At low receptor densities (i.e., *N*_*r*_ = 50, 125 in Figure S2a), the distributions are unimodal with peaks near *m* = 1 and 3, respectively, indicating poor multivalent engagement and frequent weakly bound states. As receptor density increases to *N*_*r*_ = 250 (Figure 2A), the distribution broadens with a peak at *m* ≈ 6, suggesting modest enhancement in binding. The peak increases further to *m* ≈ 11 at *N*_*r*_ = 500 but unexpectedly drops back to *m* ≈ 6 at *N*_*r*_ = 1000, likely due to steric mismatches between ligands and increasingly dense receptors. The corresponding snapshots in Figure 2A show limited membrane wrapping and minimal conformational changes, which further confirm weak avidity across conditions. Overall, the rigid NPs remain in a bound state for all receptor densities but fail to reach high multivalency due to its geometric rigidity.

**Figure 2:**
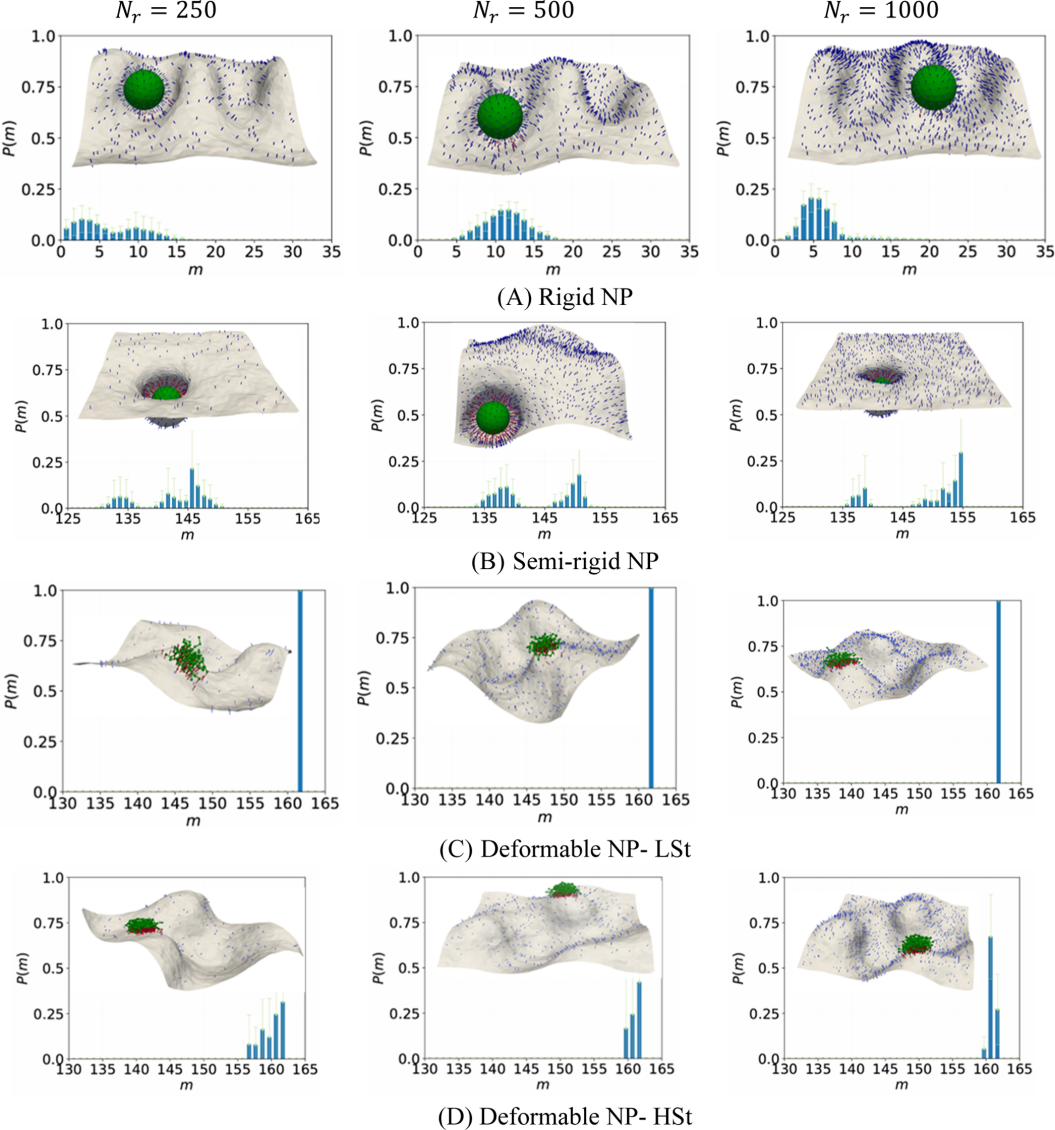
Snapshots and probability distribution of binding multivalency for different types of NPs in a strong binder system, having ligand density of ***N***_***l***_ = 162 per NP, bound to the cell membrane with different receptor densities, fixed ***A***_**ex**_ = 20% and ***k*** = 20 ***k***_***B***_ ***T***.

Semirigid NPs show significantly enhanced multivalent binding compared to their rigid counterparts, as shown in Figure 2B. At *N*_*r*_ = 50 and 125 (Figure S2b), *P*(*m*) distributions are narrow and centered around lower multivalency values (i.e., *m* ≈ 48 and 100, respectively), due to limited receptor availability. At *N*_*r*_ = 250, the distribution begins to broaden and shows early signs of bimodality. As receptor density increases to *N*_*r*_ = 500 and 1000, the distributions become distinctly bimodal with peaks around *m* ≈ 137 and 151 for *N*_*r*_ = 500, and *m* ≈ 139 and 154 for *N*_*r*_ = 1000. This bimodal behavior reflects the presence of two dominant binding states, one representing partial engagement and the other approaching full saturation. The variability arises from the mechanical interplay between the rigid NP core and flexible surface tethers, which facilitate partial membrane wrapping but allow diverse binding outcomes. This membrane wrapping phenomenon emphasizes the enhanced ability of SRG NPs to leverage increased receptor densities for more robust and extensive receptor–ligand interactions. We note that the bimodality observed for SRG NPs at high receptor density (i.e., *N*_*r*_ = 250–1000) does not arise from metastable trapping. In individual trajectories the system typically resides for extended periods in one of these modes and only occasionally switches between them on the MC time scale. As described in Numerical Setup section, each case was equilibrated and then sampled for 10^9^ MC steps in four independent replicas. Additionally, ligand–receptor bond formation/breakage were performed using configurational-bias MC with Rosenbluth weighting (as explained in SI) to improve sampling efficiency while preserving detailed balance. These simulations therefore provide extensive sampling of fluctuations within each adhesion mode, but the number of effectively independent transitions between modes remains limited. This limitation is intrinsic to the computational cost of long-time scale membrane-NP simulations and should be considered when interpreting the equilibrium multivalency distributions. Consequently, the locations of the two peaks in *P*(*m*) and the qualitative coexistence of the corresponding adhesion states are robust features of the data, whereas the relative peak heights, and thus the exact equilibrium populations of the two modes inferred from *P*(*m*), should be regarded as approximate rather than fully converged.

The LSt deformable NPs exhibit ideal multivalent binding behavior characterized by complete ligand engagement and deterministic binding distributions. Representative snapshots show a pancake-like configuration with maximum surface coverage, leading to a high degree of multivalency, regardless of receptor density. At low receptor densities (i.e., *N*_*r*_ = 50 and 125), the multivalency distributions are sharply peaked at *m* = *N*_*r*_, indicating that the ligands consistently engage all available receptors (Figure S2c). However, due to the low receptor density, some NP arms remain unbound and fluctuate freely above the membrane. From *N*_*r*_ ≥ 250, the NP saturates its own ligand capacity and sharply peaked at *m* = 162, the maximum number of ligands.

The HSt deformable NPs at *N*_*r*_ = 50 and 125 (Figure S2d) exhibits similar multivalent binding behavior as the LSt NPs, with peaks at *m* = *N*_*r*_, showing complete receptor utilization. However, as the receptor density increases beyond the ligand limit, internal mechanical resistance begins to manifest. At *N*_*r*_ = 250 and 500 (Figure 2D), *P*(*m*) broadens and peaks closer to *m* = 162, indicating that while high multivalency is achieved, structural stiffness prevents consistent saturation. Only at *N*_*r*_ = 1000 does the HSt NP consistently achieve full ligand occupancy, with the distribution collapsing into a sharp peak at *m* = 162.

We note that the aligned configurations shown for RG and deformable NPs are equilibrium free-membrane conformations, randomly sampled at 20% excess area on a finite, periodically repeated patch, that are not reshaped upon interaction with these particles. In the RG case, since binding multivalency is very low, the prebinding conformations persist. For deformable NPs, although high multivalency is achieved, it arises mainly from flattening/spreading against the membrane, which suppresses local fluctuations near the adhesion regime but does not generate additional invaginations. By contrast, for SRG NPs the rigid core with short tethers recruits curvature and induces significant wrapping, combining high multivalency with curvature recruitment and remodeling the initial configuration into a localized invagination around the NP.

In summary, the strong binder system reveals a hierarchy in binding performance shaped by NP mechanical properties. Rigid NPs remain weakly multivalent across all densities, semirigid NPs achieve high multivalency but exhibit bimodal distributions due to conformational heterogeneity, and deformable NPs (LSt and HSt) show superior binding with the LSt variant saturating consistently and early, and the HSt variant requiring higher receptor densities to overcome internal constraints.

### Multivalency of Binding for Intermediate Binder System

Figure 3 and Figure S3 (Supporting Information) summarize the results for binding multivalency of ligand-coated NPs under intermediate binder conditions, i.e., Δ*G*≈ −11.45*k*_*B*_ *T*,*k*_*b*_ = 0.38*N*/*m*. In this regime, the results demonstrate that binding is no longer universally favorable and requires mechanical or spatial assistance (e.g., NP deformability or receptor clustering) to reach significant multivalency. Rigid NPs fail to bind across all receptor densities, confirming the inability of rigid NPs to form stable interactions when binding energy is insufficient.

**Figure 3:**
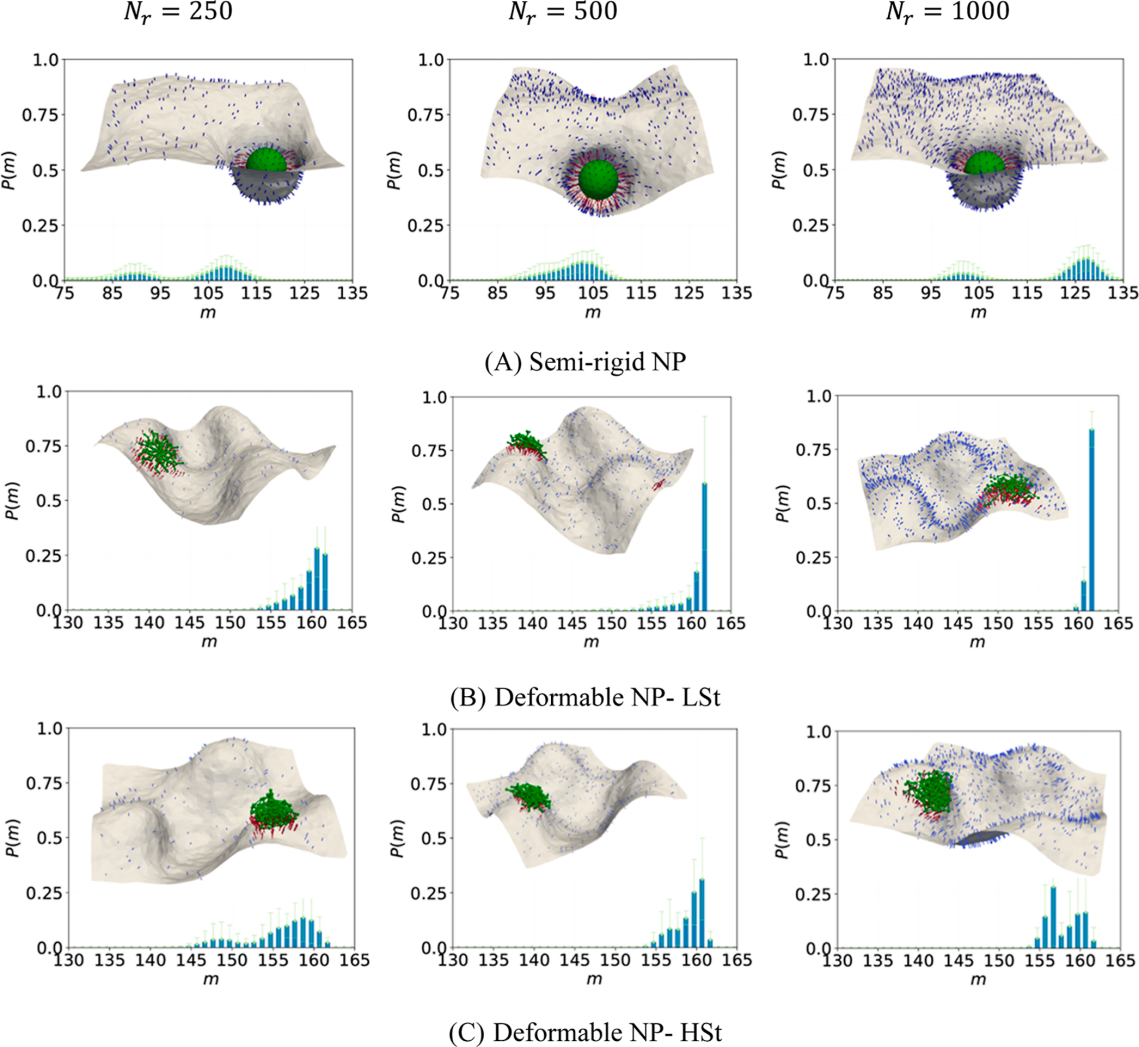
Snapshots and probability distribution of binding multivalency for different types of NPs in an intermediate binder system, having ligand density of *N*_*l*_ = 162 per NP, bound to the cell membrane with different receptor densities, fixed *A*_ex_ = 20% and *k* = 20*k*_*B*_ *T*.

SRG NPs show gradual increase in multivalency with increasing receptor density, as depicted in Figures 3A and S3a. Generally speaking, SRG NPs perform well in the intermediate regime, although their internal mechanical constraints prevent consistent saturation. At *N*_*r*_ = 50, multivalency is modest and broad, centered around *m* ≈ 26 with large variability (Figure S3a). At *N*_*r*_ = 125, the distribution shifts upward to *m* ≈ 74, indicating moderate receptor engagement. At *N*_*r*_ = 250, the NP exhibits a bimodal distribution with peaks around *m* ≈ 88 and *m* ≈ 109, suggesting a dynamic coexistence of partially and nearly fully bound states. At *N*_*r*_ = 500, the system stabilizes into a unimodal distribution around *m* = 104, reflecting uniform high multivalency. Interestingly, at *N*_*r*_ = 1000, bimodality reemerges with peaks at about *m* ≈ 102 and *m* ≈ 128, indicating that additional receptor availability can increase ligand recruitment but also reintroduces conformational diversity. The snapshots in Figure 3A show increasing degrees of membrane deformation as receptor density increases.

The LSt NPs exhibit significant binding multivalency even under intermediate affinity. At *N*_*r*_ = 50, multivalency saturates at *m* = 50, corresponding exactly to the number of available receptors. This receptor-limited saturation persists at *N*_*r*_ = 125, where *P*(*m*) increases monotonically and peaks at *m* = 125, as shown in Figure S3b. The distribution for *N*_*r*_ = 250 shows a moderately broad peak centered around *m* ≈ 158–160, with a small range extending from about *m* = 150 to the saturation limit of *m* = 162. From *N*_*r*_ ≥ 500, the distribution becomes sharply peaked at *m* ≈ 162 (the maximum number of available ligands on the NP surface), reflecting a near-deterministic binding behavior despite the intermediate binder regime. The snapshots in Figures 3B and S3b also reveal a highly flattened NP embedded in a densely populated receptor region, supporting a complete and stable multivalent interaction. HSt NPs follow a similar trend compared to LSt NPs, but display broader, noisier distributions, only reaching consistent saturation at *N*_*r*_ = 1000. The findings in Figures 3C and S3c suggest that while HSt NPs are capable of near-complete receptor engagement, internal mechanical resistance introduces structural noise and configurational diversity in the final binding state.

Overall, in the intermediate binder regime, NP flexibility becomes essential to achieving multivalency. RG NPs fail entirely to bind, confirming that mechanical deformation is necessary when ligand–receptor bonds are not intrinsically strong. SRG NPs can leverage flexible tethers to reach high multivalency, though their binding profiles are sensitive to receptor availability and mechanical variability. Both deformable NP types consistently achieve near- or full-saturation binding, with LSt NPs doing so earlier and more uniformly across all receptor densities, while HSt NPs exhibit increased configurational heterogeneity due to internal stiffness. We note that no multivalency of binding was observed for all types of NPs under weak binding conditions.

### Comparison of Average Multivalency Between Strong and Intermediate Binder Systems

To compare multivalent binding capabilities of various NP types under different ligand–receptor affinities, we analyzed the average multivalency (i.e., mean number of ligand–receptor bonds formed) against receptor densities for both strong and intermediate binder systems (Figure 4). The trend across both systems is similar, with deformable NPs consistently outperforming RG and SRG types, but with lower overall multivalency under intermediate binding.

**Figure 4:**
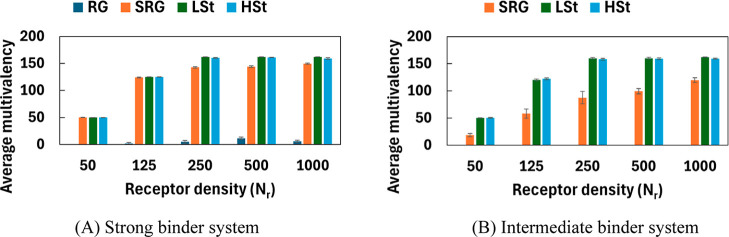
Average number of multivalent binding interactions between ligand–receptor pairs under (A) strong and (B) intermediate binding conditions, as a function of receptor density with fixed ligand density of ***N***_***l***_ = 162, ***A***_**ex**_ = 20% and ***k*** = 20 ***k***_***B***_ ***T***.

Deformable NPs remain the most effective receptor–ligand binders under both conditions. The LSt NPs achieves nearly complete saturation (≈162 bonds) as early as *N*_*r*_ = 250, maintaining strong multivalency even at moderate affinity levels. The HSt variant closely follows, although with slightly reduced values due to its internal mechanical resistance. These results are consistent with the probability distributions in Figures 2 and 3, which show sharply peaked or right-skewed distributions concentrated near full ligand occupancy. SRG NPs exhibit a similar increasing trend with *N*_*r*_ in both binder systems but reach lower multivalency under intermediate condition, consistent with the broader, bimodal distributions observed in Figure 3A. Under strong binder conditions, their average multivalency reaches a high value (≈145–150), as shown in Figure 4A. In the case of intermediate binder systems (Figure 4B), the average multivalency increases gradually with receptor density (from ≈18 at *N*_*r*_ = 50 to ≈120 at *N*_*r*_ = 1000), which illustrate their ability to leverage mechanical compliance and receptor abundance to compensate for weaker affinity. While RG NPs remain limited to low values (*m* < 15) under strong binder, even at high receptor density, they form no stable bonds in the intermediate regime, with zero multivalency across all *N*_*r*_ (Figure 4B). This confirms that mechanical flexibility is essential for binding under intermediate affinity. Our observations so far suggest that while all type of NPs benefits from increased receptor density, their ability to achieve high multivalency of binding is fundamentally governed by their deformability and the strength of ligand–receptor interactions.

### Free Energy Analysis Under Strong Binder Conditions

To assess the thermodynamic determinants of multivalent NP binding to cell membranes, we evaluated the total binding free energy (Δ*F*), along with its enthalpic (Δ*H*) and entropic (*T*Δ*S*) components, under strong and intermediate binder conditions. Figure 5 presents these thermodynamic quantities, complemented by the per receptor–ligand bond analysis (Figure S4a) as a function of receptor density. The data for all NP types reveal a clear interplay between configurational entropy and enthalpic gains that shapes the overall free energy of binding.

**Figure 5:**
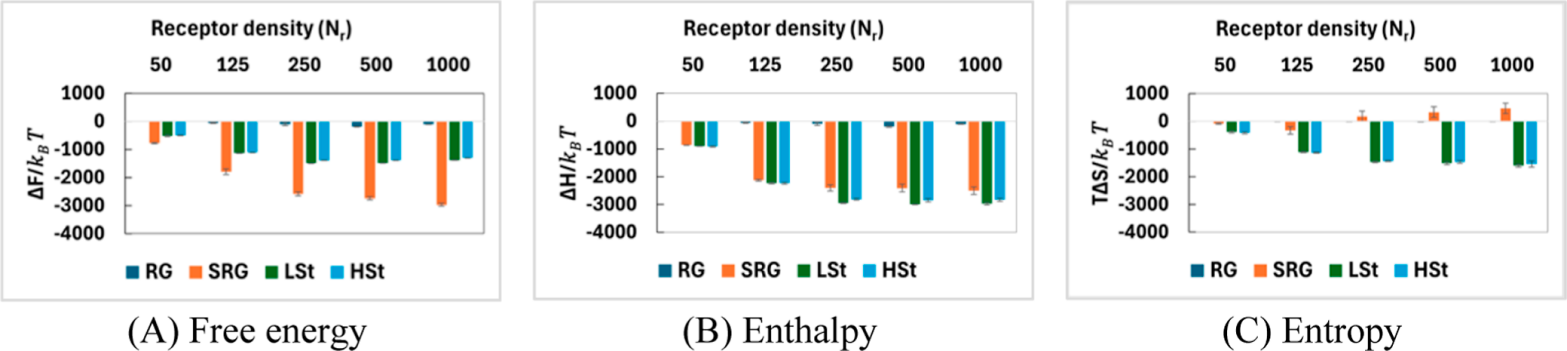
Total and per bond energy of binding in a strong ligand–receptor binder system for different NPs as a function of receptor density with fixed ***A***_**ex**_ = 20% and ***k*** = 20 ***k***_***B***_ ***T***.

Among all NP types, SRG NPs consistently exhibit the most favorable total free energy of binding (approximately 1 order of magnitude in Δ*F*/*k*_*B*_ *T*) across all receptor densities. This superior binding avidity arises from the optimal balance of strong enthalpic interactions and minimal entropic penalties. Specifically, SRG NPs demonstrated a substantial increase in enthalpy of binding, attributable to extensive multivalent binding (Figure 4A) facilitated by flexible ligand tethers and membrane remodeling.

Although binding commonly reduces configurational entropy, SRG NPs exhibit a favorable entropic contribution at high receptor densities (Figure 5C). Decomposition of the entropy change (Δ*S*) into membrane (Δ*S*_MEM_) and receptor components (Δ*S*_REC_) (Figure S5) shows that Δ*S*_MEM_ is positive and increases with *N*_*r*_, whereas Δ*S*_REC_ is negative but its magnitude decreases with *N*_*r*_. Mechanistically, the rigid-core/short-tether architecture of SRG nucleates a compact membrane invagination (wrap) when receptors are abundant. This invagination recruits local excess area at the adhesion region, relieving lateral tension and increasing the number of accessible undulation modes outside the contact region, yielding a net gain in membrane entropy. This is consistent with membrane-adhesion/undulation theory and NP-wrapping analyses.^63,64^ In parallel, high receptor availability provides many equivalently favorable binding partners within tether reach, reducing the degree of receptor localization required to maintain multivalency and thus diminishing the loss of receptor translational entropy. Consequently, the Δ*S* switches sign from negative at low *N*_*r*_ to positive at high *N*_*r*_, with the crossover occurring between *N*_*r*_ ≈ 125–250 in our simulations and becoming the most favorable at *N*_*r*_ = 1000. The per bond analysis further clarified these findings. That is SRG NPs maintained highly favorable free energy per bond, driven by moderate per bond enthalpy coupled with entropy gain per bond at elevated receptor densities (Figure S4a). Thus, individual ligand–receptor bonds formed by SRG NPs are not only thermodynamically efficient but also configurationally permissive, promoting high multivalency without incurring the configurational penalties typically observed in other NP systems.

LSt NPs also show strong total binding avidity, though consistently lower than SRG NPs across all receptor densities. The key thermodynamic characteristic for LSt NPs is their pronounced increase in enthalpy of binding, the highest observed among all NP types (Figure 5B). This significant enthalpic gain stems from highly conformal adhesion and optimal ligand–receptor pairing, aligning with their deterministic multivalency demonstrated in Figure 2C. However, the substantial enthalpic gain for LSt NPs was counteracted by considerable entropy loss, consistently observed across all receptor densities (Figure 5C). The per bond analysis further confirms this enthalpy–entropy compensation (Figure S4a). This suggests that there is a critical interplay between receptor density-induced enthalpic gains and configurational constraints. Consequently, the high total avidity of LSt NPs is primarily driven by complete ligand occupancy, despite significant configurational restrictions and reduced thermodynamic efficiency at the individual bond level.

HSt NPs exhibits a thermodynamic behavior similar to LSt NPs but with slightly low overall binding performance. Although their total enthalpy is highly favorable, it remains somewhat lower than that of LSt NPs due to internal mechanical stiffness limiting complete conformational adaptability. Similar to LSt, HSt NPs suffer considerable and consistent entropy losses at all receptor densities. Per bond analyses highlights the similarity between LSt and HSt in both enthalpy and entropy per bond, where the strong individual bond formation occurs at the cost of substantial entropy loss (Figure S4a). While increased receptor density does enhance binding strength, the stiffness-related limitations are more pronounced at moderate receptor densities (*N*_*r*_ = 250–500), indicating that optimal receptor density must balance ligand access with structural adaptability. Thus, despite individual bond strength comparable to LSt, HSt NPs show lower overall binding avidity due to restricted ligand accessibility and reduced multivalency efficiency (Figure 2D) stemming from their increased stiffness.

RG NPs demonstrate the weakest total binding free energy across all receptor densities. This is due to multivalency (Figure 2A) and modest enthalpic gains. The total entropy losses for RG NPs are smaller compared to deformable NPs but remains substantial enough to significantly weaken their net binding advantage. Notably, receptor density plays a significant role in modulating RG binding efficiency. The results show that higher receptor densities are essential to achieving even modest multivalent interactions due to their structural rigidity. The per bond analysis clarifies that, although RG NPs exhibit strong enthalpy per bond, comparable and sometimes superior to SRG NPs, their individual bonds incur modest entropy penalties and are infrequent due to structural inflexibility (Figure S4a).

In summary, the multivalent binding efficacy of functionalized NPs under strong binder conditions is critically dependent on a finely tuned balance of enthalpic gains and entropic costs, modulated by both NP flexibility and receptor density.

### Free Energy Analysis Under Intermediate Binder Conditions

The thermodynamic behavior of NP interactions with cell membranes under intermediate binder systems demonstrates notable distinctions when compared to strong binder scenarios. As shown in Figure 6, the roles of NP rigidity, deformability, and receptor density significantly influence the binding avidity of NPs. Rigid NPs with zero multivalency of binding exhibit no stable interactions across all receptor densities, which further confirm that flexibility of NPs is crucial for binding under these weaker conditions.

**Figure 6:**
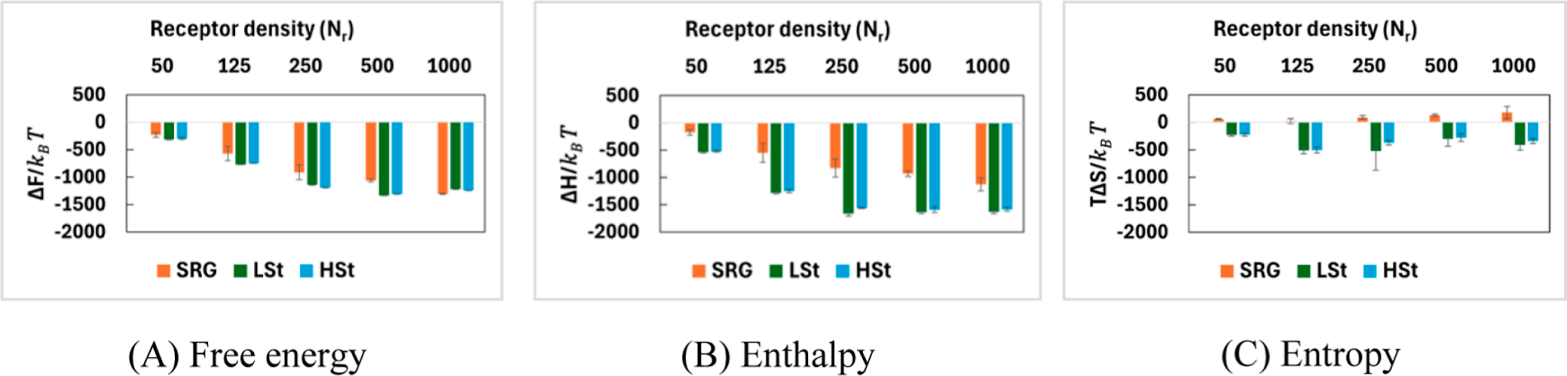
Total and per bond energy of binding in an intermediate ligand–receptor binder system for different NPs as a function of receptor density with fixed ***A***_**ex**_ = 20% and ***k*** = 20 ***k***_***B***_ ***T***.

SRG NPs demonstrate significant, yet variable, binding behaviors. Figure 6A shows that their total free energy of binding improves progressively with increasing receptor densities, becoming more favorable particularly at the highest receptor density (*N*_*r*_ = 1000). This trend results from their capacity to leverage flexible tethers and partial membrane wrapping, enhancing ligand accessibility and binding multivalency. The enthalpy data reveals that SRG NPs benefit substantially from an increase in enthalpic contributions at higher receptor densities, although less dramatically than deformable NPs. More importantly, Figure 6C shows that the entropy contribution remains relatively modest, with SRG NPs experiencing mild entropy losses even at high receptor densities. Per bond analyses (Figure S4b) further elucidate that while SRG NPs exhibit favorable per-bond free energy, their enthalpy per bond is consistently less favorable compared to deformable NPs. Interestingly, the per-bond entropy indicates minimal configurational penalties at high receptor densities. This suggests that their moderate flexibility enables effective engagement without substantial entropic cost.

LSt NPs exhibit strong binding across receptor densities, maintaining highly favorable total free energy due to their strong enthalpic contributions. However, as evident in Figure 6C, these strong enthalpic gains compete with substantial entropy losses, similar to strong binder systems. This behavior can also be observed in the per-bond analysis, where LSt NPs consistently exhibit the most favorable enthalpy per bond (Figure S4b) but also experience the greatest entropy losses per bond. Hence, their binding avidity primarily results from high receptor–ligand multivalency rather than thermodynamic efficiency per bond. HSt NPs display a behavior similar to LSt NPs, though slightly less favorable overall. Nevertheless, internal stiffness somewhat limits their conformational adaptability, reducing multivalent engagement and causing variability in binding outcomes. This stiffness introduces notable configurational entropy penalties, which, although significant, remain consistent across receptor densities.

Receptor density emerges as a vital modulator of NP binding under intermediate binder conditions. Low receptor densities significantly restrict binding efficacy, which shows the necessity of sufficient receptor density to achieve significant multivalency and binding stability. For deformable NPs, higher receptor densities substantially improve total binding avidity, with deformability effectively leveraging increased receptor availability despite entropic penalties. Semirigid NPs similarly benefit from increased receptor density but depend heavily on receptor density to shift from moderate to significant binding states.

### Comparison of Free Energy Landscape Between Strong and Intermediate Binder Systems

Comparing the thermodynamics of NP binding under strong and intermediate ligand–receptor affinity conditions reveals critical insights into how binder strength modulates multivalency and binding efficiency. Across affinities, RG NPs remain poor binders, as Δ*F* stays unfavorable or only weakly improved even as receptors increase, which reflects limited binding interactions and negligible configurational adaptability. Under strong binding, SRG designs consistently deliver the most favorable free energy over most receptor densities. They capture large enthalpic gains from stable multivalent contacts while avoiding the high deformation costs paid by highly soft particles. Deformable (LSt and HSt) NPs can match or surpass SRGs only at the highest receptor densities, where many simultaneous bindings offset their larger entropic penalties.

When affinity is reduced (intermediate regime), the balance shifts. At low-moderate receptor densities, deformable NPs outperform because their shape adaptability enables more binding per NP despite weaker bonds. As receptor density increases, SRGs gradually regain advantage. Their stiffer geometry supports sustained multivalency without excessive configurational loss, and Δ*F* becomes favorable more steeply with density than for deformable NPs. Overall, receptor density is the main selector under intermediate binding, where deformable NP is advantageous when receptors are scarce or patchy and SRG NP is favored once receptors are abundant.

### Interplay of NP Rigidity and Receptor Density on NP Avidity to the Membrane at ***A***_**ex**_ = 6%

To evaluate the role of membrane mechanical compliance in enabling multivalent NP binding to the cell surface, we examined the binding behavior of a lower excess area membrane, i.e., *A*_ex_ = 6%.

### Comparison of Binding Multivalency Between Strong and Intermediate Binder Systems

Figures 7 and 8A map the multivalency landscape under strong binding, while Figures 10 and 8B present the corresponding analysis for intermediate binding. Under strong binding, RG NP shows only modest growth of distribution of multivalency *P*(*m*) with *N*_*r*_ and remains in low-valency states across the panel (Figures 7A and S6a), consistent with limited membrane wrap and poor recruitment of additional bonds at higher *N*_*r*_. The distribution of multivalent receptor–ligand pairs for SRG NPs shifts rightward with *N*_*r*_ but do not approach saturation even by *N*_*r*_ = 1000 (Figures 7B and S6b), indicating that geometric constraints prevent full engagement at low excess area. Deformable NPs perform distinctly. LSt NP is effectively saturated once receptors are modestly available (sharp peaks at *m* ≃ 162 for *N*_*r*_ ≳ 250; Figure 7C), while HSt NP approaches saturation more gradually and often remains just below full occupancy at intermediate-to-high *N*_*r*_ (Figure 7D). These trends are reflected in the corresponding average multivalency ⟨*m*⟩ curves in Figure 8A.

**Figure 7:**
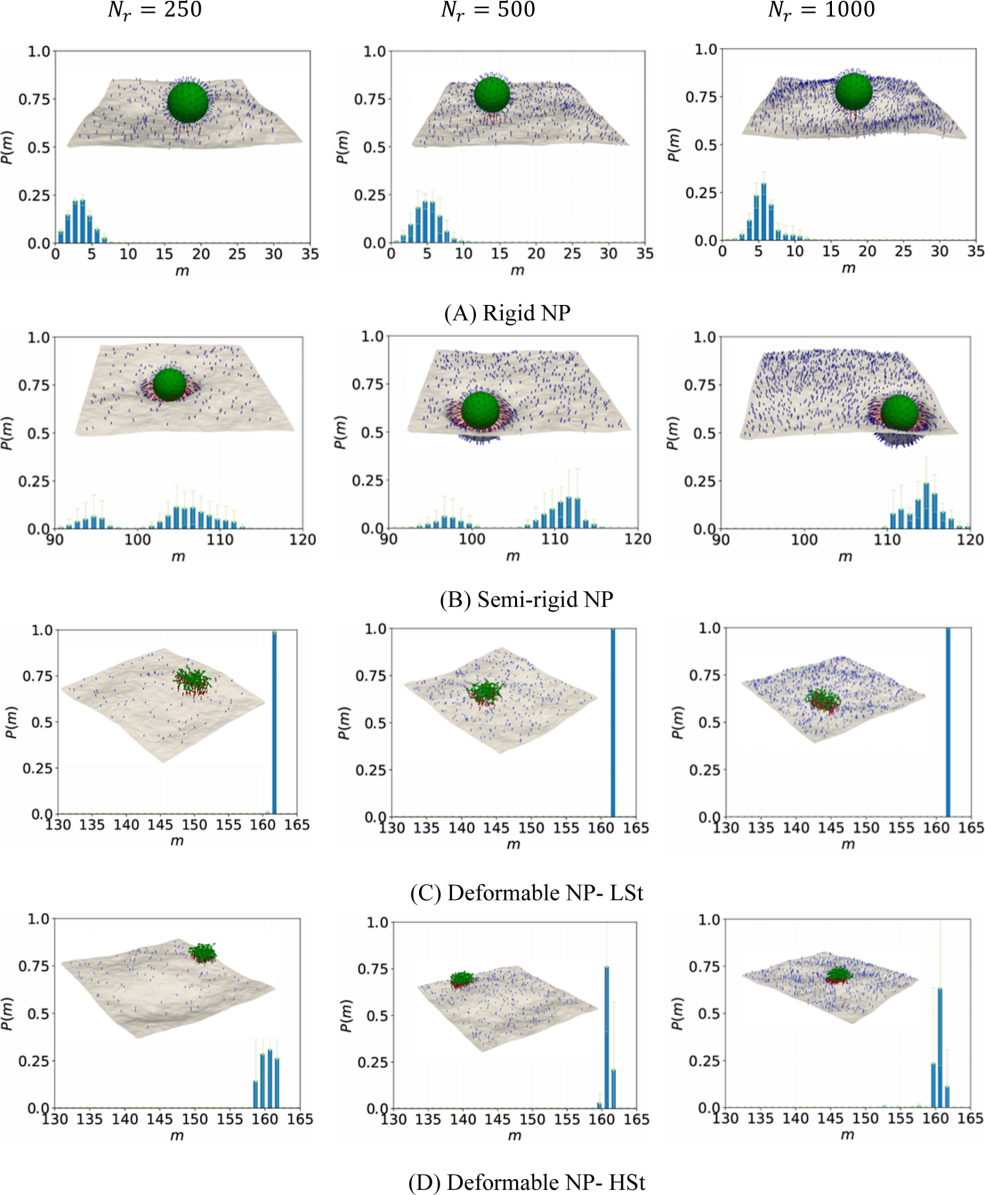
Snapshots and probability distribution of binding multivalency for different types of NPs in a strong binder system, having ligand density of ***N***_***l***_ = 162 per NP, bound to the cell membrane with different receptor densities, fixed ***A***_**ex**_ = 6% and ***k*** = 20 ***k***_***B***_ ***T***.

**Figure 8:**
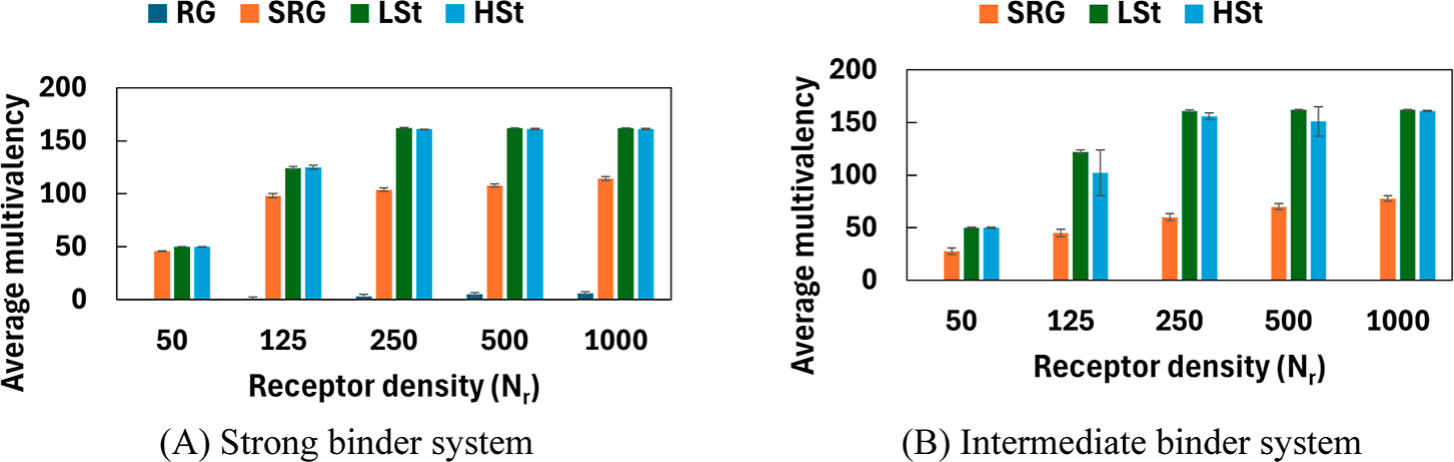
Average number of multivalent binding interactions between ligand–receptor pairs under (A) strong and (B) intermediate binding conditions, as a function of receptor density with fixed ligand density of ***N***_***l***_ = 162, ***A***_**ex**_ = 6% and ***k*** = 20 ***k***_***B***_ ***T***.

Under intermediate binding, RG NP fails to establish multivalency at any *N*_*r*_, emphasizing the need for mechanical adaptability when single-bond affinity is modest. SRG NPs retains an increasing trend with *N*_*r*_ but the *P*(*m*) peaks are left-shifted and narrower at moderate *N*_*r*_ (Figures 9A and S7a), yielding lower ⟨*m*⟩ than in the strong-binding case (Figure 8). LSt NP again exhibits near-deterministic, saturated binding by *N*_*r*_ ≈ 250, with *P*(*m*) sharply peaked at the ligand limit and ⟨*m*⟩ flat at *m* ≃ 162 thereafter (Figures 9B and 8B). The distribution of binding multivalency for HSt NP at *N*_*r*_ = 125–250 broadens and its peak flattens, whereas at higher *N*_*r*_ the system reaches saturation yet remains more dispersed than LSt NP, indicating heterogeneous engagement even when receptors are abundant (Figure 9C).

**Figure 9:**
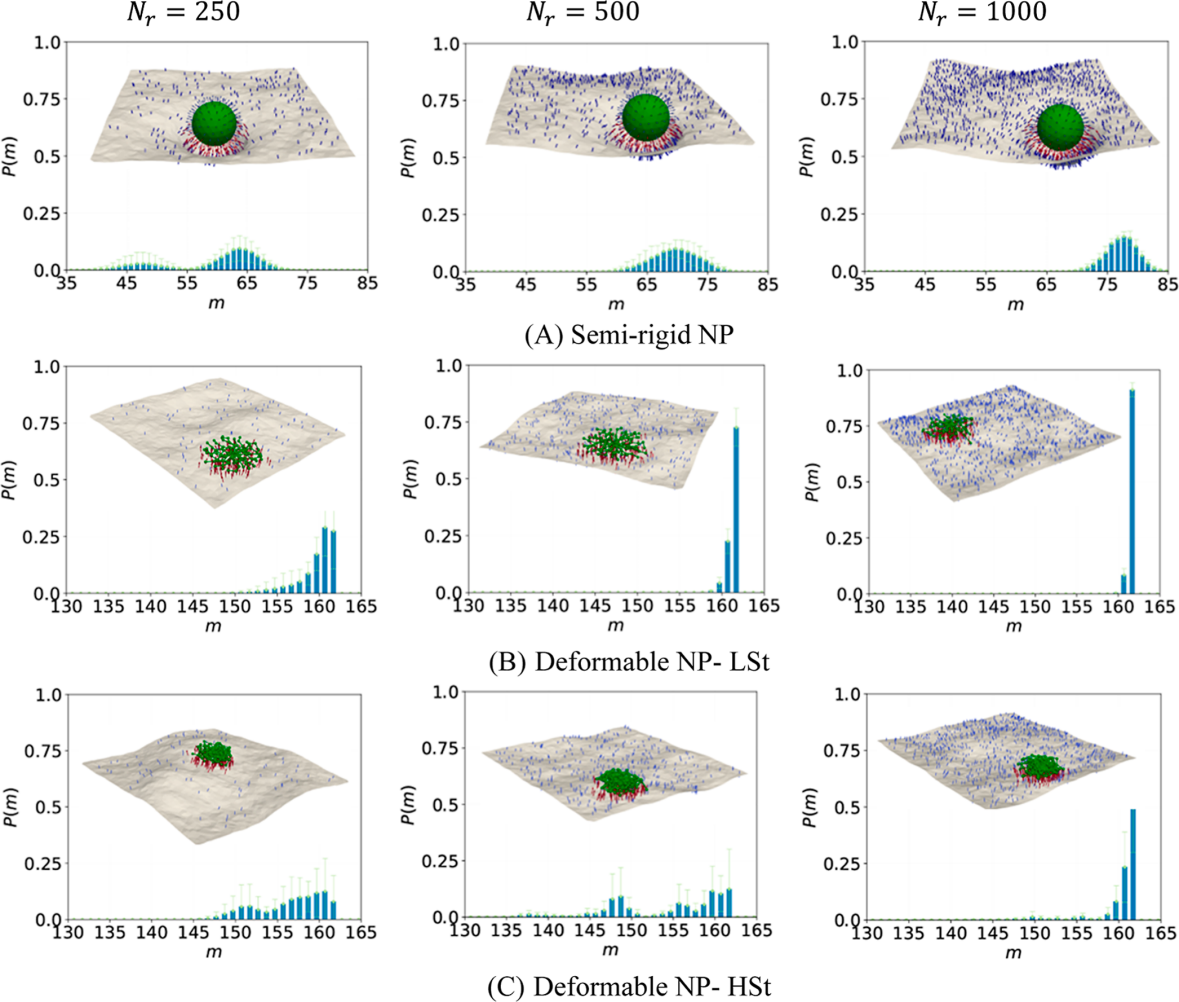
Snapshots and probability distribution of binding multivalency for different types of NPs in an intermediate binder system, having ligand density of ***N***_***l***_ = 162 per NP, bound to the cell membrane with different receptor densities, fixed ***A***_**ex**_ = 6% and ***k*** = 20 ***k***_***B***_ ***T***.

Moving from strong to intermediate affinity at low excess area of membrane creates a clear affinity gap in multivalency for RG and SRG NPs across the full *N*_*r*_ range, while deformable NPs retain high engagement, with LSt largely saturated and HSt close but more variable (Figures 7–9). In short, when excess area is limited, mechanics and affinity are partially substitutable: deformability of LSt and HSt NPs can sustain high multivalency even at intermediate affinity, SRG NP relies more on affinity (especially at low-to-moderate *N*_*r*_), and RG NP is ineffective without strong bonds.

### Comparison of Binding Energy Landscapes Between Strong and Intermediate Binder Systems

To directly observe how monovalent affinity modulates multivalent adhesion at low membrane excess area, we compare the free-energy landscapes in Figure 10 for free energy of binding and Figure S8 for enthalpy and entropy of binding, respectively. At *A*_ex_ = 6%, Figure 10 shows that increasing monovalent affinity systematically increases the total free energy of adhesion across all NP types and receptor densities, with strong binders consistently more favorable than intermediate binders at matched *N*_*r*_, consistent with per-bond analysis in Figure S9. This shift with affinity and receptor density is largely enthalpy-driven, with the entropy term depending on NP mechanics (Figure S8).

**Figure 10:**
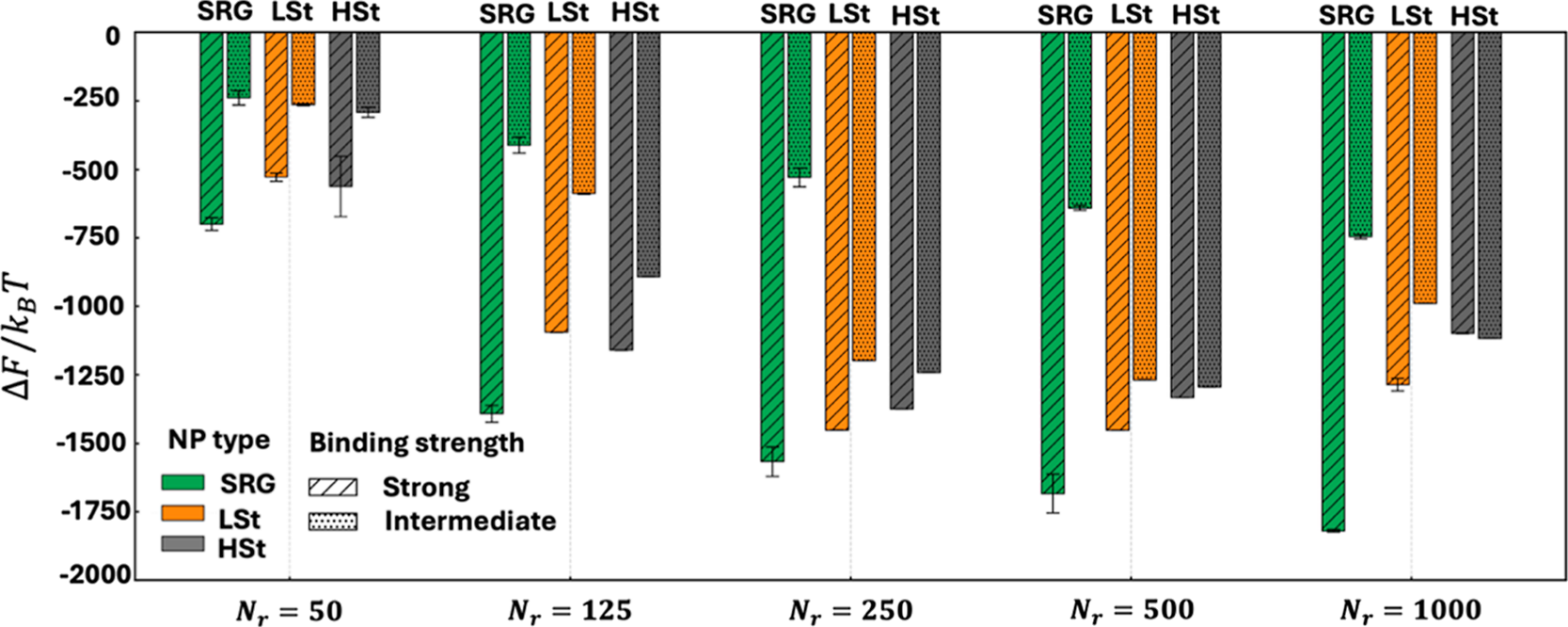
Comparison of free energy of binding between intermediate and strong ligand–receptor binder systems for different NPs as a function of receptor density with fixed *A*_ex_ = 6% and *k* = 20*k*_*B*_ *T*.

For SRG, strong binding produces a monotonic increase in Δ*F* with *N*_*r*_, consistent with a substantial enthalpic gain that outperforms a smaller entropic cost as receptors become abundant. In contrast, intermediate binding improves more slowly with *N*_*r*_ because weaker single-bond interactions at low excess area hinder the stabilization of compact adhesion patches. The net effect is a clear strong-intermediate gap over the full receptor-density range at *A*_ex_ = 6%. LSt and HSt display a different balance. Even under intermediate binding, Figure 10 shows comparatively favorable Δ*F* that approaches saturation at high *N*_*r*_. Figure S8a clarifies that the deformability of NPs enables extensive, conformal adhesion and receptor engagement, yielding large enthalpic gains that persist regardless of affinity. The trade-off is entropic as *T*Δ*S* remains strongly negative. This suggests that the benefit of stronger affinity for deformable NP, while present, is smaller in relative terms than for SRG at the same *N*_*r*_.

We also note that, RG NPs in intermediate binding, fail to establish stable adhesion (essentially zero multivalency), while under strong binding it achieves only modest gains that remain weak overall. Therefore, the free-energy gap between strong and intermediate is large, but both are comparatively unfavorable. This resonates with our multivalency analysis where RG requires both abundant receptors and membrane compliance to achieve even moderate engagement, conditions that are not met at *A*_ex_ = 6%.

Together, these comparisons at *A*_ex_ = 6% show that affinity and mechanics are partially substitutable levers for avidity, but not uniformly across designs. SRG leans more heavily on affinity to overcome geometric and membrane constraints when excess area is limited, whereas deformable NPs rely on enthalpy from widespread contact to maintain favorable Δ*F* even with intermediate affinity, accepting a persistent entropic cost in return.

### Mechanics-Affinity Phase Diagrams

Here we organize the results into mechanics-affinity phase diagrams on the (*A*_ex_, *N*_*r*_) plane to show where strong adhesion, multivalency, and receptor sensitivity arise jointly from NP stiffness and bond strength. We summarize each state point with three linked quantities: the adhesion free energy Δ*F*, the fractional coverage θ formed in equilibrium, and the local selectivity index α. The ligand-normalized fractional coverage θ = ⟨*m*⟩/*N*_*l*_^*tot*^ reports multivalency (how many bonds are engaged on average ⟨*m*⟩), relative to total NP ligands *N*_*l*_^*tot*^ = 162. The selectivity index α = *d* ln*θ*/*d* ln *N*_*r*_ measures how sharply multivalency θ responds to number of receptors *N*_*r*_ on a cell.^37^ This allows us identify design windows where deep free-energy minima, robust bond engagement, and selective responses coincide, and understand how these windows shift with membrane excess area and receptor density.

Figure 11 presents the phase map for the strong binder system. First, the adhesion free energy Δ*F* is shown by tile color with more negative Δ*F* identified as more favorable binding. Second, we display ligand-normalized fractional coverage θ as a gray strip whose darkness increases with receptor–ligand bond pairs, and a white dot is placed when θ crosses a fixed reference level θ* = 0.5. This half-coverage threshold is a midengagement marker, where beyond θ = 0.5, the system is typically in a robustly bound regime. Third, we visualize the selectivity index α as a narrow orange bar at the right edge of each tile, drawn only where θ is neither near zero nor saturated (i.e., 0.1 ≤ θ ≤ 0.9). The internal tick on the bar corresponds to α = 1. When the local slope reaches or exceeds this level, we add a white triangle to mark a superselective response (α ≥ 1). When 0 <α < 1, the response is enhanced but sublinear.^37^

**Figure 11:**
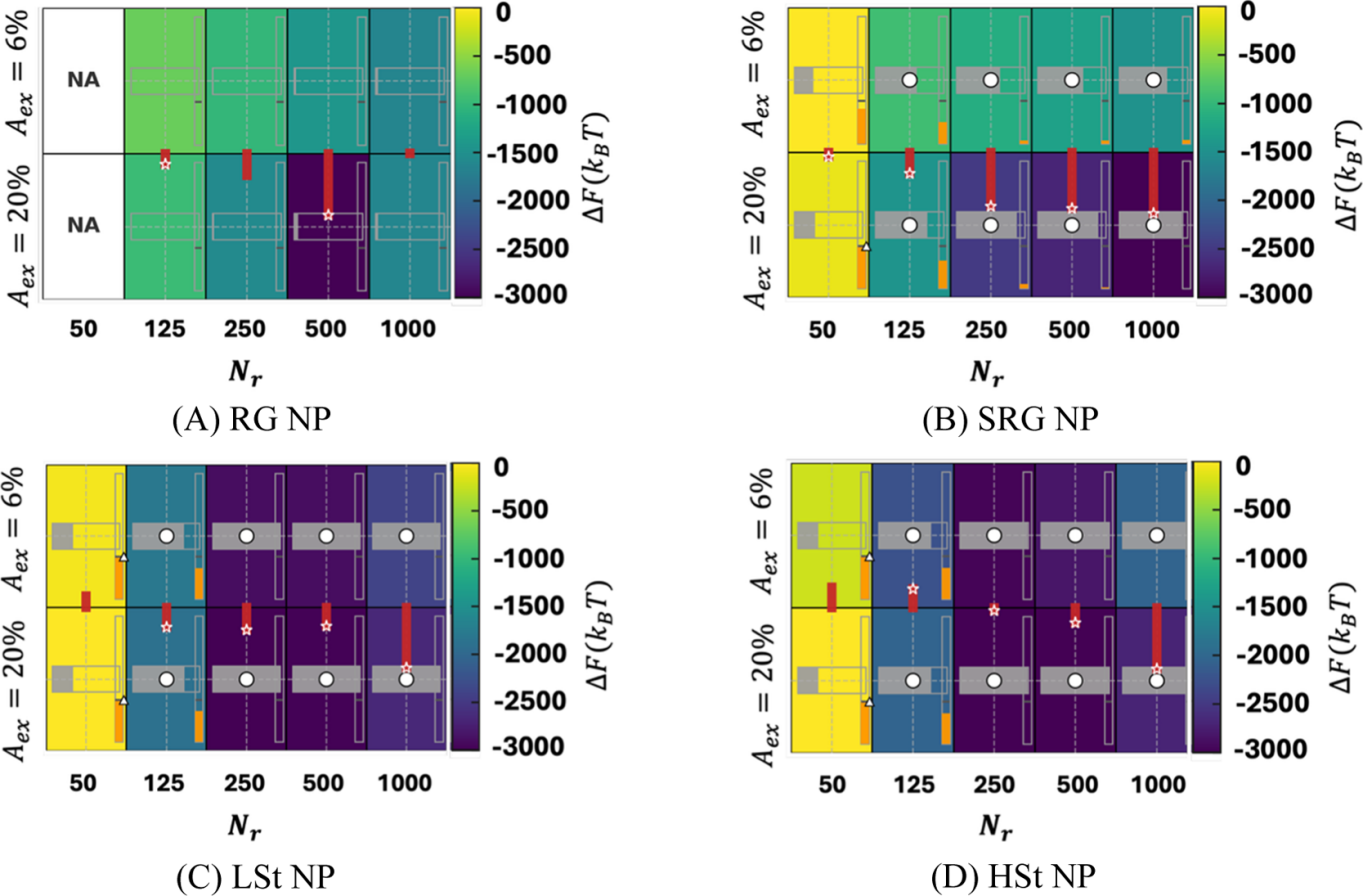
Mechanics–affinity phase diagrams for strong binders on the (***A***_ex_, ***N***_***r***_)­grid. Each tile reports three quantities for a given (***A***_ex_, ***N***_***r***_): (i) tile color = Δ ***F*** (darker = more negative); (ii) gray strip = ligand-normalized fractional coverage **θ** = ⟨ ***m*** ⟩/***N***_***l***_^***tot***^ with a white dot (○) at **θ** ≥ θ* = 0.5; (iii) orange bar at the tile’s right = local selectivity **α** = *d* **lnθ**/*d* **ln*N***_***r***_, evaluated within a nonsaturated **θ** window; the internal tick is **α** = 1 and a white triangle (Δ) marks **α** ≥ 1­(superselective). For each ***N***_***r***_, the crimson connector compares Δ ***F*** (20%) vs Δ ***F*** (6%) and points toward the more favorable ***A***_ex_; star marker (☆) at the connector tip denotes an FDR-corrected significant difference (***q*** < 0.05; Welch test). Deformable NPs (LSt, HSt) show superselective onsets at ***N***_***r***_ = 50 for both ***A***_ex_; SRG exhibits a brief superselective onset at ***N***_***r***_ = 50 for ***A***_ex_ = 20%; RG shows no superselective tiles.

Because a major question is how membrane excess area changes binding at fixed receptor density, we also place a crimson connector between the two *A*_ex_ rows at each *N*_*r*_. The connector points toward the row with the more favorable free energy. A white star at the connector tip indicates that the difference in Δ*F* between *A*_ex_ = 6% and 20% is statistically significant. Significance is assessed with Welch’s two-sample test, which produces a *p*-value. Because we test many (*A*_ex_, *N*_*r*_) contrasts, we control for multiple comparisons using the Benjamini–Hochberg procedure.^65^ This yields *q*-values, which are *p*-values adjusted to limit the expected false discovery rate (FDR). In the figure, a star means *q* < 0.05 for that contrast. The underlying *p*-values for these row-to-row Δ*F* comparisons are reported in Tables S2 and S3, and the starred markers in the figure correspond to those contrasts that remain significant after FDR correction.

At fixed *N*_*r*_, increasing excess area from *A*_ex_ = 6% to 20% generally deepens Δ*F*. This effect is strongest for SRG NPs, where Δ*F* (20%) is more favorable than Δ*F* (6%) at every *N*_*r*_ and all contrasts are significant, consistent with the enthalpy gains we observe when increasing *A*_ex_ allows the membrane to wrap and stabilize a compact adhesion patch. For the deformable NPs (LSt, HSt), the advantage of increasing *A*_ex_ becomes clear once receptors are not limiting, i.e., for *N*_*r*_ ≥ 125, whereas at *N*_*r*_ = 50 the difference is small or absent. The multivalency of binding follows these thermodynamic tendencies. LSt and HSt NPs maintain midto-high θ across much of the (*A*_ex_, *N*_*r*_) grid, and the strong multivalency (θ marker) appears for *N*_*r*_ ≈ 125 and above at both *A*_ex_, indicating robust engagement once receptors are modestly available. SRG reaches θ*at the same *N*_*r*_ ≈ 125 for both *A*_ex_, but θ is higher (darker gray) at 20% than at 6% at the same *N*_*r*_, showing that excess area increases multivalency of binding.

For deformable NPs, superselective onsets (delta marker) occur at low receptor density (*N*_*r*_ = 50) at both excess-area levels. As *N*_*r*_ increases, the fractional coverage θ approaches saturation and the response transitions from superselective to enhanced (i.e., the orange bar shortens). For SRG NPs, the selectivity is enhanced at low *N*_*r*_ and can become superselective at *A*_ex_ = 20% and *N*_*r*_ = 50. RG NP shows little leverage from excess area across *N*_*r*_, in line with its weak avidity, and neither robust multivalency nor strong selectivity is observed across the grid.

Practically, the diagram predicts that membrane flexibility and NP deformability shift binding into the high-θ, favorable-Δ*F* regime and advance the onset of selective responses to lower *N*_*r*_. By contrast, SRG designs require both higher *N*_*r*_ and more excess area to achieve similar avidity, and RG NPs are ineffective under the same conditions.

Figure 12 shows that moving from strong to intermediate affinity narrows the design window for superselectivity and shifts emphasis from “how much the membrane can spread” to “whether increasing excess area can be converted into” additional concurrent bonds. Across NP types, Δ*F* becomes more favorable with receptor density, but the leverage of excess area is weaker and more selective than in the strong-binding maps.

**Figure 12:**
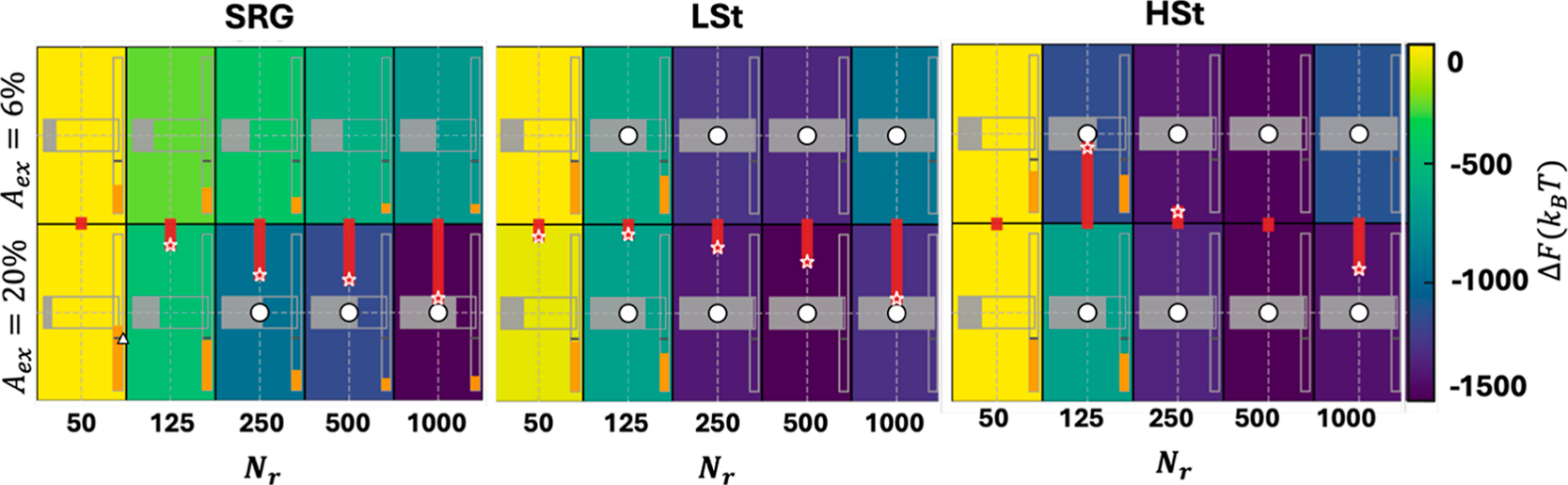
Mechanics-affinity phase diagrams for intermediate binders on the (*A*_ex_, *N*_*r*_) grid.

SRG shows the clearest area dependence. for *N*_*r*_ ≥ 125 the crimson bar points to *A*_ex_ = 20% and is marked as significant difference (star markers) from *N*_*r*_ ≥ 25, in line with the deeper Δ*F* at higher excess area. The fractional coverage (gray strip) brightens steadily with *N*_*r*_, yet θ* is reached only along the 20% row (dot markers) and only once receptors are abundant (*N*_*r*_ ≥ 250). At *A*_ex_ = 6% the system remains subthreshold throughout. Selectivity (orange bar) is concentrated at the low-density edge, where responses are enhanced (0 < α < 1) but not superselective in the valid window (i.e., *N*_*r*_ = 50–125).

LSt maintains high multivalency across the grid even at intermediate affinity, where θ*is crossed from *N*_*r*_ ≈ 125 at both excess-area levels, and increasing *A*_ex_ mainly broadens, not shifts, the high-θ band (gray stripe). Free-energy contrasts favor 20% for *N*_*r*_ ≥ 125 (starred connectors), reflecting enthalpic gains that outweigh the entropy cost once receptors are not limiting. The orange bar is again largest at *N*_*r*_ = 50–125 and short thereafter, with selectivity index being enhanced but not superselective under intermediate affinity (no delta marker). HSt NP mirrors LSt in overall but shows one notable difference. The starred crimson connector flips toward *A*_ex_ = 6% at *N*_*r*_ = 125 despite darker tiles at 20% elsewhere. This crossover is consistent with a transitional regime in which extra excess area promotes NP spreading without yet recruiting enough additional bonds for a soft, but not ultrasoft, NP. The added spreading area raises membrane entropic costs faster than enthalpy grows, so Δ*F* (6%) is briefly more favorable. Second, once *N*_*r*_ increases, the usual 20% advantage returns and is significant at *N*_*r*_ = 1000. As with LSt NP, the θ*dot appears from *N*_*r*_ ≈ 125 at both rows and the orange bars are strongest at low *N*_*r*_ without triangles, indicating enhanced but not superselective responses under intermediate affinity.

Two broader differences with the strong-binding maps are worth emphasizing. First, superselective onsets at low *N*_*r*_ that appear for deformable NPs under strong binding do not occur for intermediate binder systems. This is due to the fact that weaker per-bond lifetimes smooth the θ­(*N*_*r*_) rise for LSt and HSt, keeping α < 1 in the dynamic window even though multivalency becomes robust at moderate *N*_*r*_. SRG, by contrast, can display a superselective onset at *A*_ex_ = 20%, *N*_*r*_ = 50 under both strong and intermediate affinity, where the coverage remains subthreshold at 6% and only gradually crosses θ*at *A*_ex_ = 20%. Second, the free-energy advantage of *A*_ex_ = 20% is more conditional in the intermediate case. It is strongest and most statistically secure for SRG and for deformable NPs at higher *N*_*r*_. However, at very low *N*_*r*_ the rows are similar and, for HSt NPs near *N*_*r*_ = 125, the sign can invert as discussed above.

## Conclusion

In this study, we employed a statistical mechanics based computational framework complemented by thermodynamic analyses to systematically examine the multivalent binding interactions of ligand-functionalized NPs with cell membranes. By varying NP type (rigid, semirigid, and deformable), ligand–receptor affinity (strong and intermediate binding regimes), and receptor densities, we revealed context-dependent mechanisms governing NP binding and adhesion efficiency.

### Role of NP Rigidity

Our analysis clearly demonstrates the critical role of NP rigidity in modulating multivalent binding interactions. Rigid NPs consistently showed the lowest binding efficiency across all tested conditions, limited primarily by their inability to adaptively conform to membrane receptor distributions. Conversely, semirigid NPs with polymeric tethers exhibited significant binding advantages under conditions of strong ligand–receptor affinity and higher receptor densities, primarily driven by substantial enthalpic gains. However, deformable NPs with tunable stiffness outperformed semirigid variants under intermediate affinity conditions or lower receptor densities by effectively utilizing their configurational flexibility to maximize receptor engagement despite significant entropic penalties.

### Effect of Receptor Density

Receptor density emerged as a pivotal modulator of NP binding performance. At low receptor densities, NP multivalency was severely constrained, highlighting the importance of sufficient receptor clustering for achieving stable adhesion. Notably, deformable NPs effectively leveraged higher receptor availability, significantly enhancing their binding avidity under intermediate binding conditions. Semirigid NPs demonstrated a pronounced dependence on receptor density, requiring higher receptor densities to transition from moderate to strong binding states. Thus, receptor density critically dictates the interplay between NP structural flexibility and ligand–receptor affinity in multivalent interactions.

### Effect of Membrane Mechanics

The mechanical properties of cell membranes, specifically their deformability (excess area or tension), significantly impacted NP binding. Reduced membrane compliance notably decreased multivalent interactions, especially hindering rigid and semirigid NPs due to limited receptor accessibility caused by suppressed membrane wrapping. In contrast, deformable NPs maintained robust multivalent engagement even when membrane mechanics were restrictive, which indicates their intrinsic advantage in adapting to mechanical constraints.

### Entropy–Enthalpy Compensation

Thermodynamic analyses revealed the importance of balancing enthalpic gains from ligand–receptor interactions against entropic penalties associated with restricted configurational freedom of the NPs and receptors. Semirigid NPs efficiently minimized entropy penalties while achieving considerable enthalpic contributions, particularly under strong affinity conditions. On the other hand, deformable NPs experienced larger entropy costs due to their extensive conformational changes, especially evident when approaching saturation binding conditions. These contrasting entropic behaviors emphasize the necessity of carefully tuning NP flexibility in the context of intended biological applications.

### Comparative Performance Across Binding Strengths

Comparisons between strong and intermediate ligand–receptor affinity scenarios highlighted critical trade-offs. Semirigid NPs excelled under strong affinity conditions due to efficient enthalpy-driven multivalency but experienced diminished performance at lower affinities, particularly at reduced receptor densities. Deformable NPs displayed increased relative performance under intermediate conditions, leveraging their mechanical adaptability to engage more receptors despite weaker individual bonds. Thus, NP flexibility and receptor density interplay intricately, dictating binding avidity across varying ligand–receptor affinity landscapes.

### Mechanics–Affinity Mapping

Phase diagrams reveal that NP deformability, especially on membranes with high excess area, shifts adhesion toward strong binding, broad surface coverage, and sharp receptor sensitivity at lower receptor density. SRG NPs require higher receptor density or membrane excess area, and RG NPs rarely reach these states. In effect, mechanics can partly substitute for affinity. When affinity weakens, deformability preserves favorable adhesion and narrows, rather than eliminates, the selective window.

Finally, while our equilibrium framework does not account for kinetic rates (e.g., rates of adhesion, wrapping, or internalization) and molecular complexity such as lipid diversity and membrane proteins, it captures the essential physical determinants of multivalent adhesion and membrane remodeling. These simplifications define the scope of our mechanics-affinity maps, which serve as predictive tools for how NP stiffness and membrane properties govern binding outcomes. However, in biological contexts, particularly in regimes where adhesion is thermodynamically borderline, differences in the time scales of NP transport, receptor mobility, and membrane remodeling may delay or suppress adhesion behaviors relative to equilibrium predictions.^66^ Future work will extend this framework by coupling the thermodynamic landscapes to kinetic models and incorporating molecular heterogeneity to address time-dependent behaviors and applicability in complex cellular environments.

In summary, this comprehensive computational exploration elucidates critical design principles for optimizing NP stiffness and flexibility in targeted therapeutic and diagnostic applications. By highlighting the complex interactions between NP mechanics, receptor density, membrane properties, and receptor–ligand affinity, our findings provide foundational insights into enhancing the selective targeting efficiency of functionalized nanocarriers.

## Supplementary Material



## Notes

Additional analysis for the interplay of NP stiffness, membrane deformation, bond strength, and receptor density on multivalent adhesion between ligand-coated nanoparticles and cell membrane receptors (PDF)
