# Supplementary material for: From Single Ligand–Receptor Bond Strength to Collective Avidity: Mechanics-Guided Superselective Nanoparticle Adhesion to Biological Membranes
Source: Langmuir. 2025 Dec 16;41(51):34210–26. doi: 10.1021/acs.langmuir.5c03244 (PMC12756914; doi:10.1021/acs.langmuir.5c03244)
Supplement: Supplementary file 1 [file la5c03244_si_001.pdf]

Supporting Information

From single ligand-receptor bond strength to collective avidity: mechanics-guided superselective nanoparticle adhesion to biological membranes

Morteza Hamzeh, Saba Mirahsani, Fatemeh Ahmadpoor, Samaneh Farokhirad*

Department of Mechanical and Industrial Engineering, New Jersey Institute of Technology, Newark, NJ 07114, USA

Table of Contents

- *Supplementary Methods*
- *Supplementary Figures*
- *Supplementary Tables*

* Correspondence: samaneh.farokhirad@njit.edu

Supplementary Methods

Multiscale model for multivalent receptor-ligand interactions

To investigate the binding interactions between ligand-coated nanoparticles (NPs) and receptors diffusing on cell membranes, a multiscale computational model is utilized [1, 2]. This model integrates equilibrium statistical mechanics with continuum field representations of cell membranes and coarse-grained molecular models for ligands, receptors, and deformable NPs. This approach allows for the study of multivalent binding, where multiple ligand-receptor pairs interact simultaneously to form stable adhesion complexes. The model captures the essential physical mechanisms and interactions, including NP translation and rotation, receptor translation and flexure, ligand-receptor bond formation, membrane mechanics and fluidity, and changes in configurational entropy, in terms of energy functionals and interaction potentials.

We compute the binding free energy as $\Delta F = F_{bound} - F_{unbound} = \Delta H - T\Delta S$. The enthalpy of binding (ΔH) is the ensemble average of different components (Eq. S5) accumulated in Monte Carlo simulations, which includes membrane deformation, NP internal interactions, receptor flexure, and ligand-receptor bond energies. The entropy change (ΔS) is the sum of three configurational terms computed from the same trajectories: NP entropy by quasiharmonic (QH) of bead fluctuations, membrane entropy by the same harmonic-oscillator (QH) approach on vertex fluctuations, and receptor translational entropy using the 2D Sackur-Tetrode expression from receptor root-mean-square fluctuations. This framework follows and extends our prior work [1]. The individual entropy and enthalpy components are described below.

Model for receptors on membrane surface. Each participating receptor in the receptor-ligand multivalent interaction is modeled as a flexible rod of total length L_r with a flexural angle θ_i with respect to its unflexed state. The total flexural energy is given by [3]:

$$H_{flex} = \sum_{i=1}^{N_r} \frac{1}{2} k_{flex} (L_r sin\theta_i)^2 \tag{S1}$$

Here k_{flex} is the flexural stiffness of the receptor. Eq. (S1) is activated only when a receptor is bonded to a ligand.

Model for the cell membrane. The cell membrane is taken as a square patch of dimension $\mathcal{L}$ and is chosen as a set of N_m nodes, T_m triangles, and L_m links to form a triangulated surface. The elastic energy of the membrane H_{memb} is modeled using a discrete form of the Helfrich Hamiltonian model [4], which accounts for the bending rigidity k and surface tension σ and is given by:

$$H_{memb} = \sum_{v=1}^{N_m} \left[\frac{\kappa}{2} \left(c_{1,v} + c_{2,v} \right)^2 + \sigma A_v \right], \tag{S2}$$

where, $c_{1,v}$ and $c_{2,v}$ are the two principal curvatures and A_v is the curvilinear area associated with vertex v. Since membrane conformation has a fixed projected area, i.e., $A_p = \mathcal{L}^2$, any nonzero value of the membrane excess area A_{ex} accommodates the additional area through undulations and

is defined by $A_{ex} = 100 \times (A - A_p)/A$. Here, A is the total curvilinear area of the membrane expressed as $A = \sum_{v=1}^{N_m} A_v$. The common range of bending rigidity and excess area for endothelial cells is $20 - 200\ k_B T$ [5] and 10-30% [3] based on the lipid composition.

Models for distinct types of NPs. We model three types of NPs, namely rigid, semi-rigid, and deformable NPs. The model for rigid NP is a sphere with targeting ligands loaded directly on its surface at a density of $N_l = 162/NP$. The model for the semi-rigid NP is the same as that of the rigid NP, except the ligands are attached to the NP surface via polymeric tethers. We modeled the tethers using a freely-jointed-chain (FJC) model by assuming a tether elasticity. The parameters of the semi-rigid NP model are available in [1] and presented in Table 1. For deformable NPs, due to deformations occurring within their structure, the energy associated with these structural rearrangements should be considered in the total energy of the system. Our focus is on a core-shell polymeric NP consisting of a lysozyme rich core with a dextran-rich corona, capable of hosting guest molecules, including small hydrophobic drugs and contrast imaging agents [6, 7]. Such core-shell nanostructures can be investigated for a range of biotechnology and biomedical applications involving diagnostic imaging and therapeutic delivery [6]. The bead-spring model is used to model the core-shell NPs and the stiffness of the springs is modeled by the FJC model [1], i.e., $k_s = \frac{3k_BT}{N_k b_k^2}$ with N_k and b_k being the number of Kuhn's segments per bead and the size of each Kuhn's segment, respectively. The microstructure of core-shell NPs is modeled as 25 arms attached to the core, verified experimentally [8], with each arm having four beads connecting adjacently through bonds [9]. The NP stiffness in the model can be tuned by introducing additional harmonic interactions between the beads (crosslinks). Here, we consider a low-stiffness NP with no crosslinking (CL) density (i.e., CL0) which has an elasticity modulus of $0.43\ kPa$ [9] and a high-stiffness NP with CL=160 which has an elasticity modulus of $15\ kPa$ [9]. All beads of the core-shell NPs interact by their adjacent connected beads through a harmonic potential, while the Weeks-Chandler-Anderson potential is used for excluded volume interactions between beads [10]. The total interaction energy of the NP is then given by:

$$H_{NP} = \sum_i \sum_j \frac{k_s}{2}(r_{ij} - r_0)^2 + \sum_i \sum_j 4\varepsilon \left[\left(\frac{\sigma}{r_{ij}}\right)^{12} - \left(\frac{\sigma}{r_{ij}}\right)^{6} + \frac{1}{4} \right] \tag{S3}$$

where, $r_{ij} = |r_i - r_j|$ is the distance between two interacting beads i and j, $\varepsilon = 0.7k_BT$ is the interaction strength, σ is the radius of the excluded volume, and r_0 is the equilibrium bond distance which is set to $2a$, with a being the bead radius.

Model for receptor-ligand bond. The NP is functionalized using ligand molecules specific to target receptors on the membrane surface. The ligand-receptor binding energy is modeled using the Bell bond potential, which is a quadratic function of the bond length and is given by [11]:

$$H_{bond} = \sum_{i=1}^{m} \Delta G_0 + \frac{1}{2} k_b^{eff} (d - d^{eq})^2 \tag{S4}$$

The distance between the receptor and ligand pair at binding site, d, is compared to a cutoff distance d^*, beyond which no binding occurs. Eq. S4 accounts only for the binding contributions of m receptors, where ΔG_0 represents the energy barrier's magnitude, distinguishing the bonded state from the unbonded state. d^{eq} is the equilibrium distance for binding interaction, which is equal to $d^{eq} = \sigma/2 + L_l$, where L_l is ligand length. d^* is the distance that binding can occur in its range, which is defined by $d^* = \left(-\frac{2\Delta G_0}{k_b^{eff}}\right)^{1/2} + d^{eq}$.

The way we define the effective stiffness of receptor-ligand bond depends on the type of NPs. For the case of rigid NP, it is simply the stiffness of the bond spring formed between the pairs of receptors and the ligands, i.e., $k_b^{eff} = k_b$. In the case of semi-rigid and deformable NPs, the effective bond stiffness includes two springs in series: one is for ligand-receptor bond (k_b) and one is for the stiffness of the tethers in semi-rigid or stiffness of the links between beads in deformable NPs (k_s), i.e., $k_b^{eff} = \frac{k_s . k_b}{k_s + k_b}$.

The total relative energy of binding for NPs bound to deformable membranes with m multivalent receptor-ligand bonds is the sum of the receptor flexure energy, ligand-receptor binding energy, membrane energy and interaction energy of deformable NP, i.e.,

$$\Delta H_{total} = \Delta H_{flex} + \Delta H_{memb} + \Delta H_{bond} + \Delta H_{NP} \quad \text{(S5)}$$

This energy formulation enables us to assess the overall stability of the multivalent binding system and distinguish configurations that are dominated by enthalpic contributions (favorable binding).

Monte Carlo Simulation

To sample the conformational states of the NP-membrane system at thermal equilibrium, we use the Metropolis Monte Carlo (MC) method that consists of six independent moves: (1) a standard MC move [12] for thermal fluctuations of membrane through vertex move, where randomly selected node on the triangulated surface is moved to a new position [13], (2) a standard MC move for membrane fluidity through link-flip process, where a randomly selected link in the membrane is replaced by a new link [13], (3) a standard MC move for NP translation, (4) a standard MC move for NP rotation, (5) a standard MC move for random diffusion of receptors [14, 15], (6) a configurational bias MC move using the Rosenbluth sampling technique [16] for flexure of receptors [14, 15], and (7) a configurational bias MC move using the Rosenbluth sampling technique for formation and breakage of receptor-ligand bonds. Such sampling reserves detailed balance and thus no biasing potentials were applied. For a detailed description of the various MC moves, see earlier publications [1, 3]. It must be noted that the NP translation to a new position for the case of deformable core-shell NPs, is accomplished through the use of equations of motion using Brownian dynamics to evolve the beads of NPs [1]. The position of all beads will be updated during this step based on both Brownian and non-Brownian forces. Non-Brownian forces include both intermolecular interactions of all beads (F_i^{NP}), which includes bead-to-bead interaction and bead-to-bead repulsion, and receptor-ligand binding interaction (F_i^b):

$$\frac{\Delta r_i}{\Delta t} = \frac{F_i^{Br} + F_i^{NP} + F_i^b}{\xi_i} \quad \text{(S6)}$$

where, $\xi_i = 6\pi\mu a$ is the friction factor. The Brownian forces (F_i^{Br}) are considered as white noise and given by the following expression:

$$\begin{aligned} &< F_i^{Br}(t) > = 0 \\ &< F_i^{Br}(t)F_j^{Br}(t') > = 6k_B T\xi_i\delta_{ij}\delta(t-t')\mathbf{I} \end{aligned} \quad \text{(S7)}$$

where, $\mathbf{I}$ and $\delta(t-t')$ are the second-order identity tensor and Dirac delta function, respectively. At each step, a random MC move is proposed for the system, while all other variables remain fixed. The acceptance of the attempted move is determined according to $P_{acc} = \min(1, \exp(-\beta\Delta H))$ [12, 16]. Here, β is defined as $(k_B T)^{-1}$, where k_B is the Boltzmann constant and T is the absolute temperature. The total energy difference, ΔH, between the bound and unbound states is computed using the energy terms defined in Eq. S5.

Free energy analysis

To quantify the overall free energy associated with NP binding to the cell surface, essential to assess the binding avidity, it is necessary to calculate the cost in the configurational entropy and the gain in binding enthalpy within the system. The enthalpy of binding is computed as $\langle\mathcal{H}\rangle$, with the ensemble averages of $\mathcal{H}$ in Eq. S5 computed during the MC simulation run. Both ligands and receptors possess significant translational entropy due to their unrestricted movement, much of which is expected to be lost upon specific binding. Another major contributor to entropy loss is the deformable core-shell NP itself, particularly the entropy reduction of the polymeric NP's constituent beads. Consequently, while multivalent receptor-ligand binding results in an enthalpic gain (i.e., Eq. S4), it also induces a loss of configurational entropy, which may counterbalance the enthalpic contribution. Using a recently developed thermodynamic model [1], we quantify the entropic contributions arising from fluctuations in the center-of-mass positions of the deformable NP beads, the motion of membrane vertices, and the spatial fluctuations of all receptors.

Entropy of deformable NP. According to the quasiharmonic analysis [17], the fluctuations in the motion of NP beads are approximated as independent harmonic oscillators. The principal component analysis of positional fluctuations of NP beads is used to compute the configurational entropy of flexible NPs [18, 19], which is recorded in the MC trajectory files:

$$S_{NP} = k_B \sum_i [\frac{h\omega_i/k_B T}{\exp(h\omega_i/k_B T) - 1} - \ln[1 - exp(-h\omega_i/k_B T)] \quad \text{(S8)}$$

where, h is the Planck's constant divided by 2π, and ω_i is the set of angular frequencies linked to eigenvalues through the equipartition theorem. When computing the quasiharmonic entropy, it's important to note that three of the eigenvalues pertain to translational movement around the center of mass, another three eigenvalues are associated with rotational motion around the center of mass, and the remaining eigenvalues, totaling 3N-6, correspond to vibrational motions. The

quasiharmonic entropy can be calculated from the angular frequencies (ω_i) derived from the system's motion.

Entropy of the membrane. For the membrane, similar to the deformable NP, we use MC trajectory files of the membrane vertices fluctuations and compute the entropy of the membrane through the harmonic oscillator model (i.e., Eq. S8).

Entropy of receptors. The translational entropy of diffusing receptors is calculated using the Sackur-Tetrode equation [20]. This method assumes that receptors behave as a two-dimensional ideal gas and do not interact with each other in a dilute solution. The equation is used to estimate the entropy of N_r receptors by considering the root-mean-square fluctuations of their center of mass. The Sackur-Tetrode equation for the behavior of an ideal gas made up of indistinguishable molecules with a mass of is defined as:

$$S_{receptor} = k_B \left[\sum_{i=1}^{N_r} \ln \left(\frac{2\pi m k_B T}{N_r h^2} \sigma_{xi} \sigma_{yi} \right) + 2 \right] \quad \text{(S9)}$$

where, x_i and y_i are the principal root-mean-square fluctuations for the center of mass of receptor i. Overall, the total entropy of the system can be computed as $\Delta S = \Delta S_{NP} + \Delta S_{memb} + \Delta S_{receptor}$.

Supplementary Figures

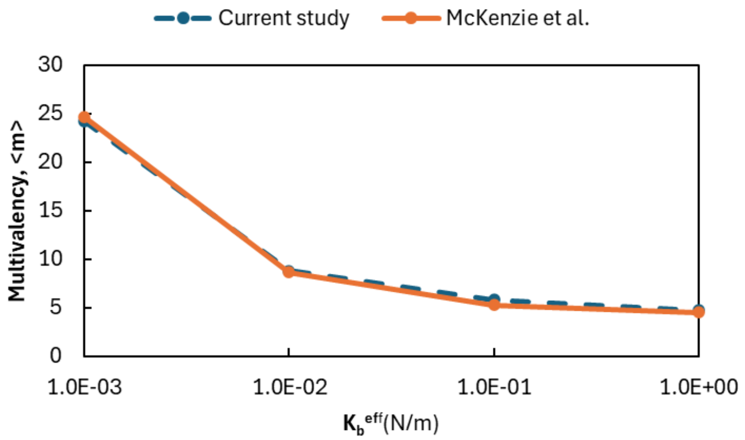

Figure S1. Validation of rigid-NP model under flat, mean-field compliant membrane assumptions: Mean multivalency $\langle m \rangle$ as a function of the effective stiffness $\frac{k_{eff}=k_b k_m}{(k_b+k_m)}$ for a rigid NP interacting with a membrane. Our simulations reproduced the multivalency trends reported by McKenzie *et al.* [23] over the range $k_{eff} \in [10^{-3}-1]N/m$.

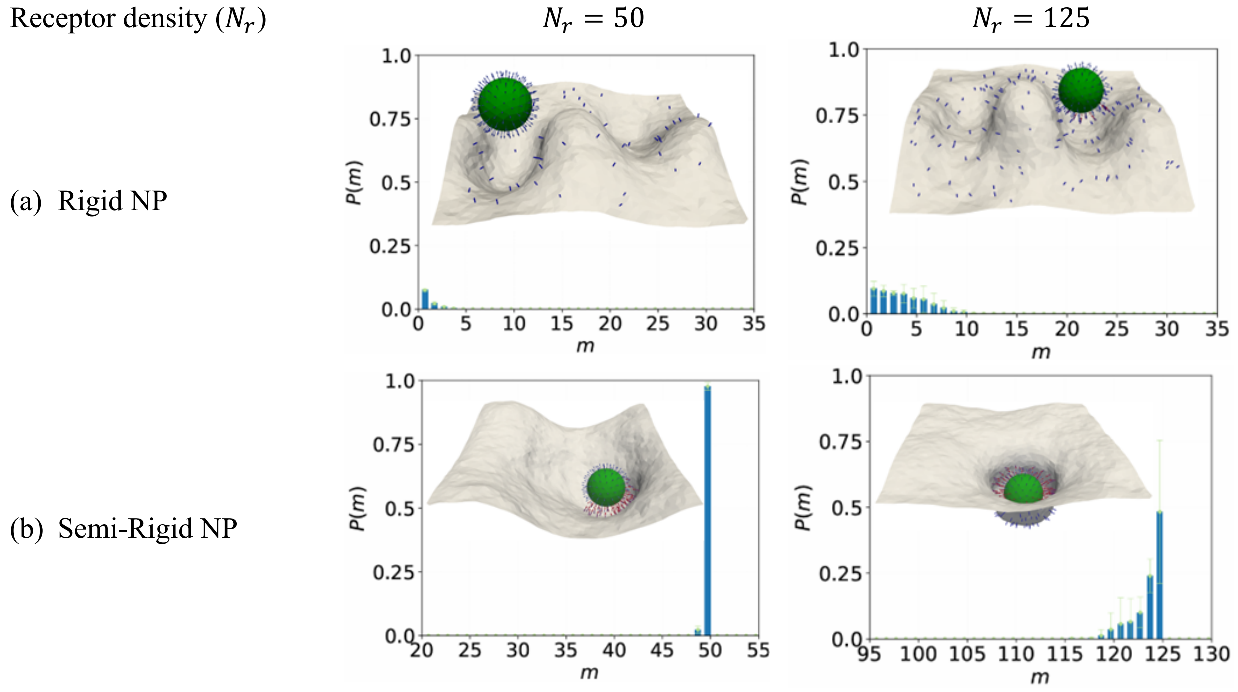

(c) Deformable NP- LSt

(d) Deformable NP- HSt

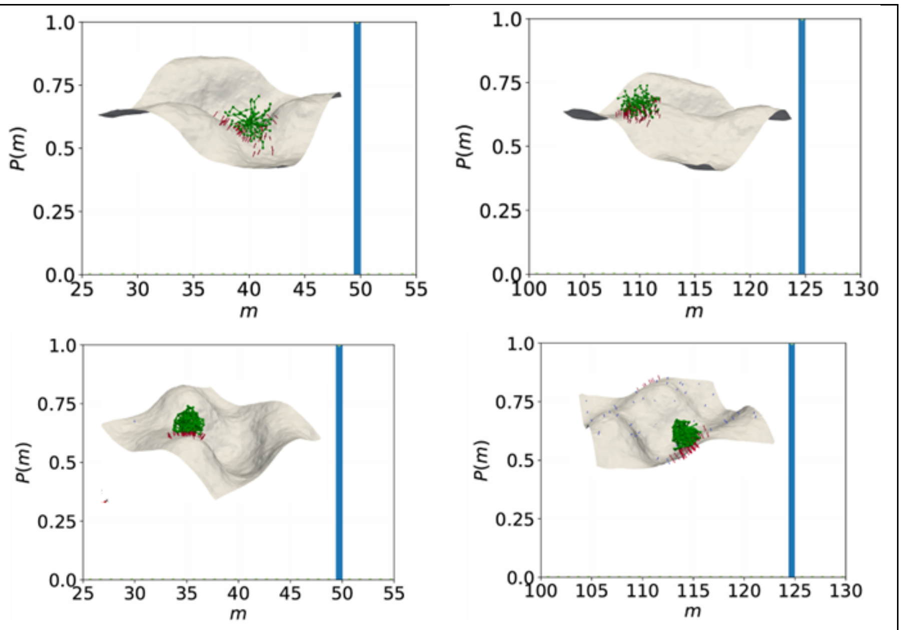

Figure S2. (a)-(d) The equilibrium distribution of the number of simultaneous receptor-ligand bonds in a strong binder system for rigid (RG), semi-rigid (SRG), and deformable (LSt and HSt) NPs with $N_l = 162/NP$ and membrane excess area of $A_{ex} = 20\%$, as functions of receptor density.

Receptor density (N_r) $N_r = 50$ $N_r = 125$

(a) Semi-Rigid NP

(b) Deformable NP- LSt

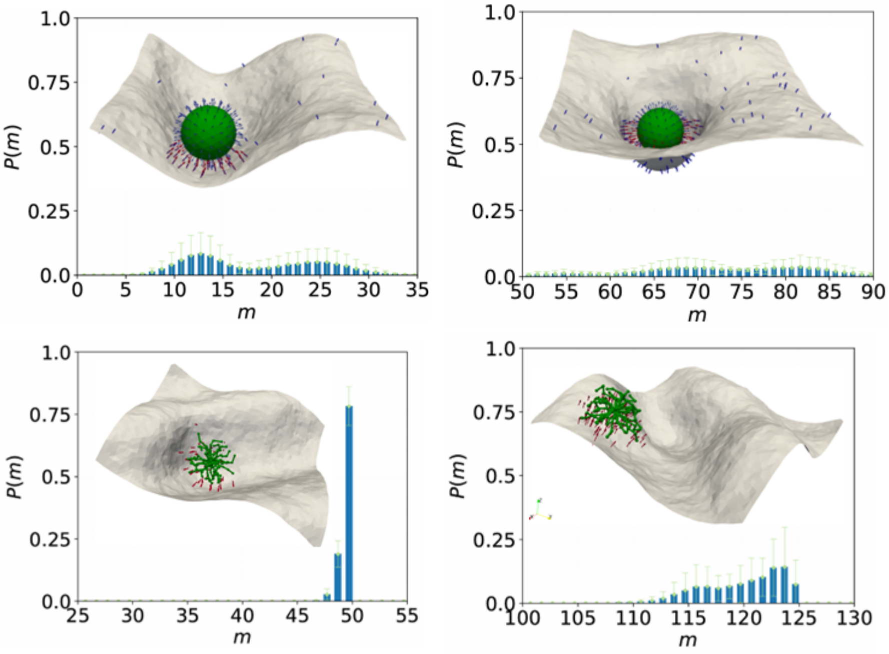

(c) Deformable NP- HSt

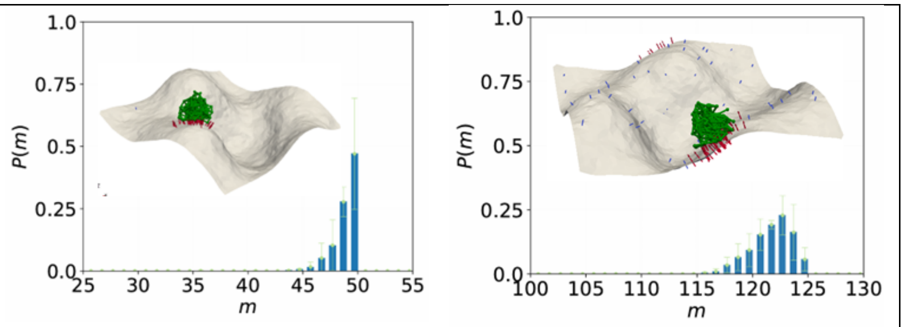

Figure S3. (a)-(c) The equilibrium distribution of the number of simultaneous receptor-ligand bonds in an intermediate binder system for semi-rigid, and deformable (LSt and HSt) NPs with $N_l = 162/NP$ and membrane excess area of $A_{ex} = 20\%$, as functions of receptor density.

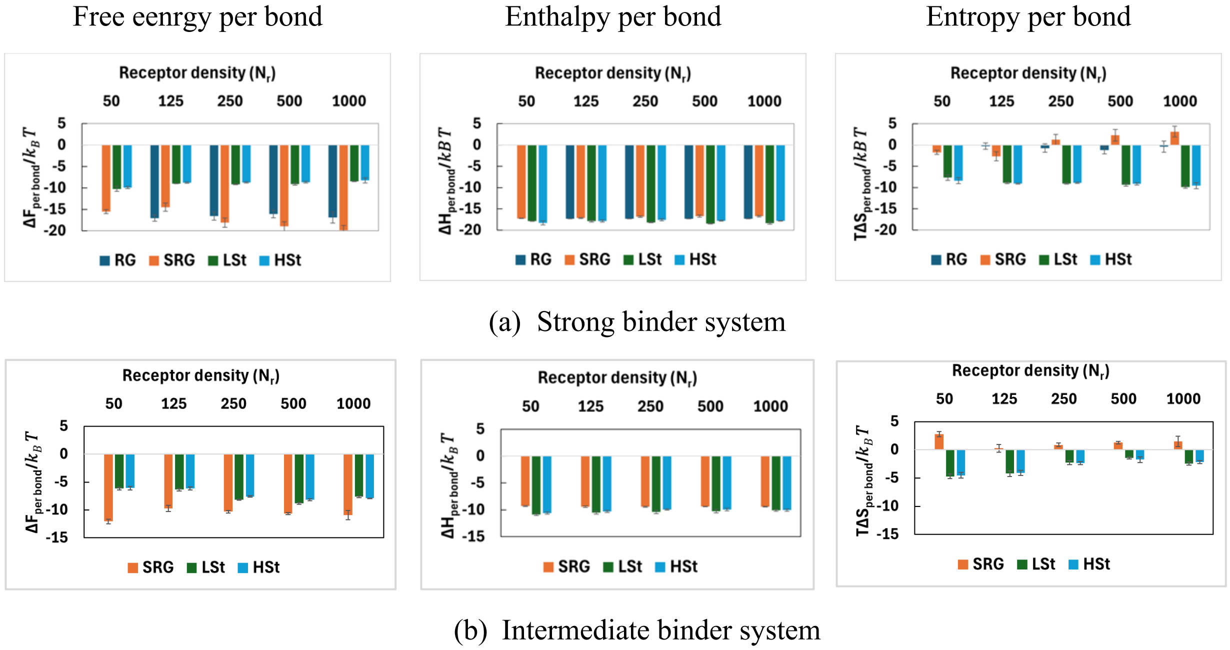

Figure S4. Relative free energy (ΔF), enthalpy (ΔH), and entropy ($T\Delta S$) of binding per receptor-ligand bond under (a) strong and (b) intermediate binder systems for different NP types as a function of receptor density with fixed $A_{ex} = 20\%$ and $k = 20k_BT$.

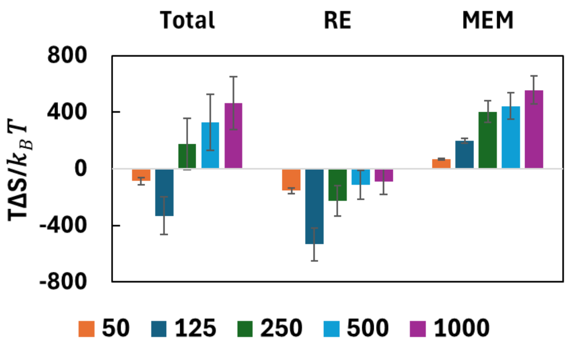

Figure S5. Components of entropy change ($T\Delta S$) of a semi-rigid NP bound to a membrane with $A_{ex} =$ 20%. RE, MEM in the figure denote receptor and membrane, respectively.

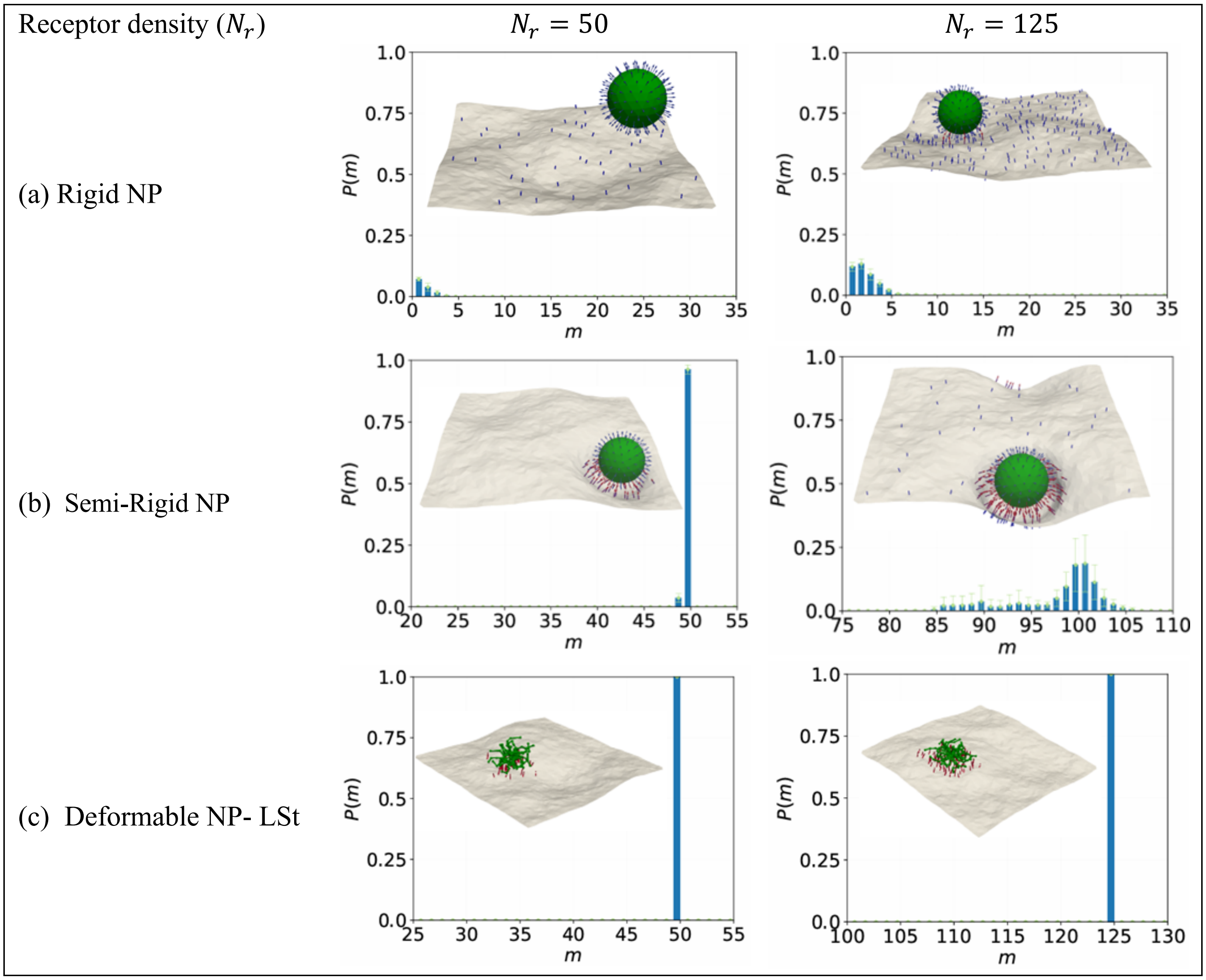

(d) Deformable NP- HSt

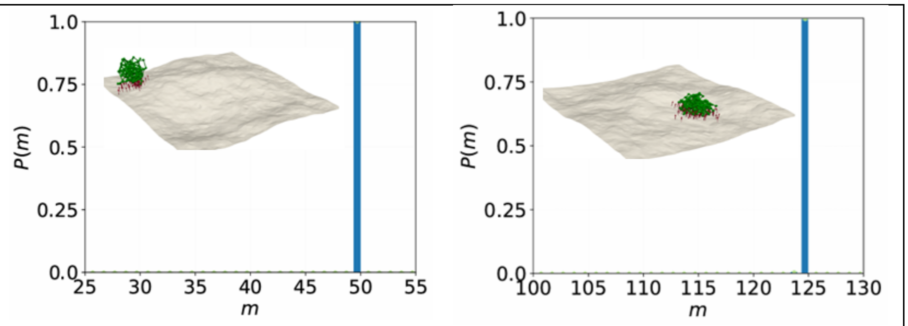

Figure S6. (a)-(d) The equilibrium distribution of the number of simultaneous receptor-ligand bonds in an strong binder system for rigid, semi-rigid, and deformable (LSt and HSt) NPs with $N_{ab} = 162/NP$ and membrane excess area of $A_{ex} = 6\%$, as functions of receptor density.

Receptor density (N_r) $N_r = 50$ $N_r = 125$

(a) Semi-Rigid NP

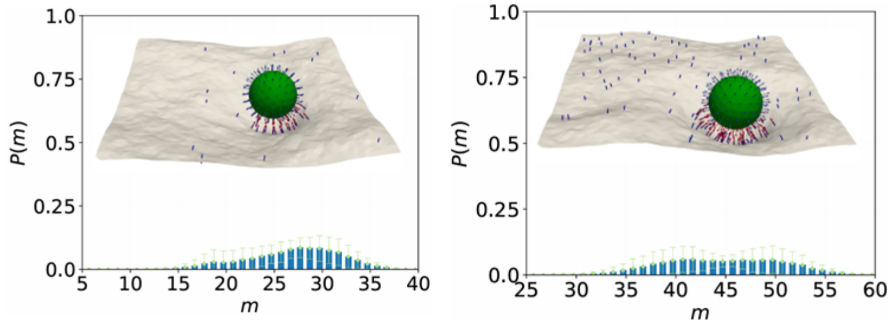

(b) Deformable NP- LSt

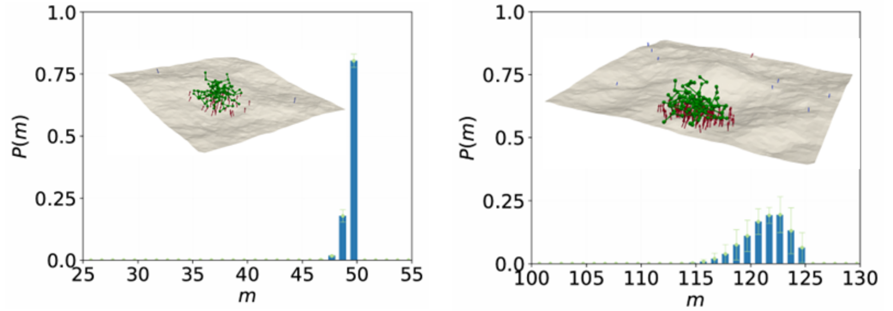

(c) Deformable NP- HSt

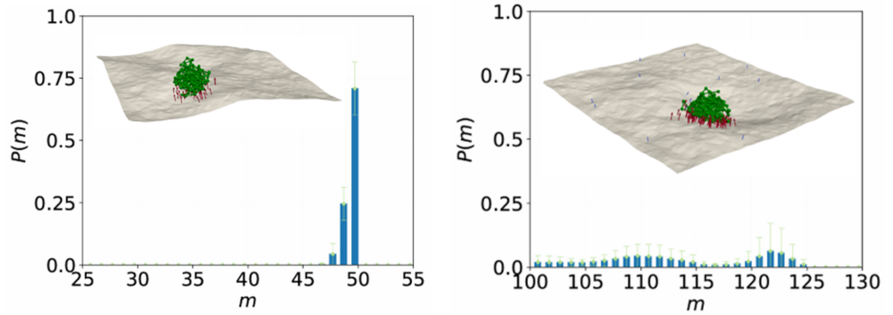

Figure S7. (a)-(c) The equilibrium distribution of the number of simultaneous receptor-ligand bonds in an intermediate binder system for semi-rigid, and deformable (LSt and HSt) NPs with $N_{ab} = 162/NP$ and membrane excess area of $A_{ex} = 6\%$, as functions of receptor density.

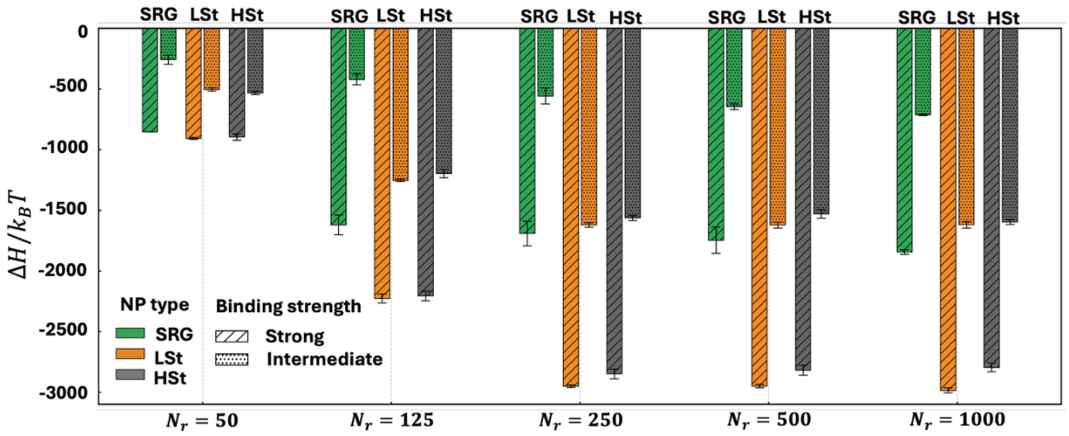

(a) Enthalpy

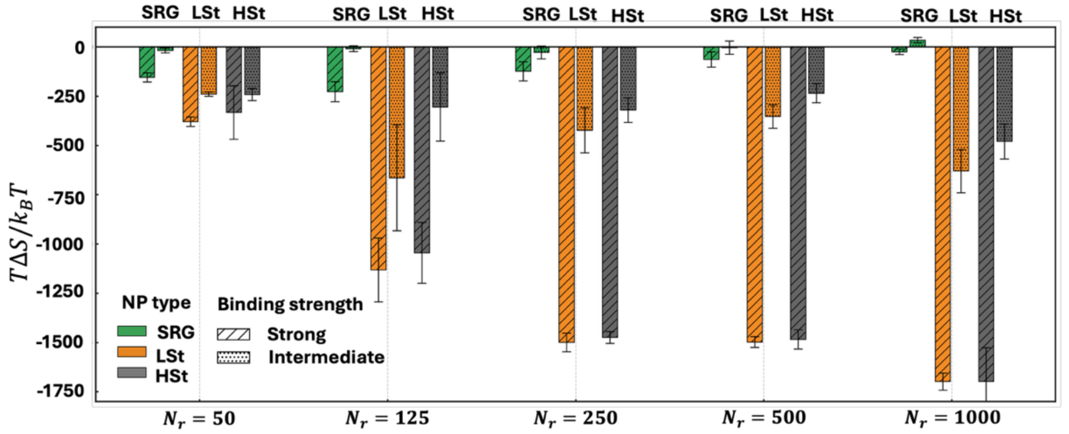

(b) Entropy

Figure S8. Comparison of (a) enthalpy and (b) entropy of binding between intermediate and strong ligand-receptor binder systems for different NPs as a function of receptor density with fixed $A_{ex} = 6\%$ and $k = 20k_BT$.

Free eenrgy per bond | Enthalpy per bond | Entropy per bond

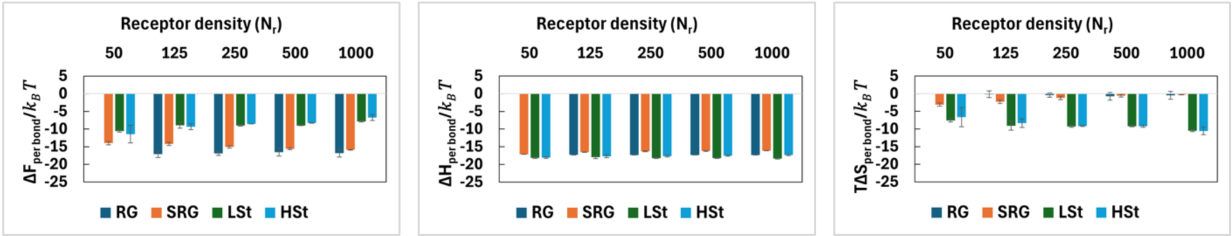

(a) Strong binder system

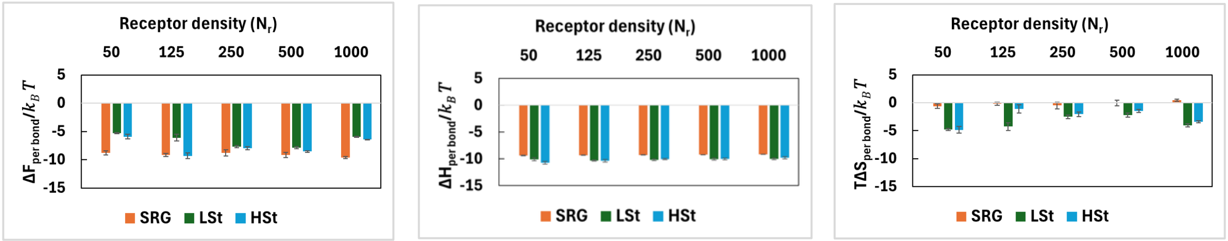

(b) Intermediate binder system

Figure S9. Relative free energy (ΔF), enthalpy (ΔH), and entropy ($T\Delta S$) of binding per receptor-ligand bond under (a) strong and (b) intermediate binder systems for different NP types as a function of receptor density with fixed $A_{ex} = 6\%$ and $k = 20k_BT$.

Supplementary Tables

NP/cell surface type	Methods	Findings and citation
deformable/Rigid	CGMD & MC/*in vivo*, *in vitro*	Shear-enhanced binding, effects of RBC volume fraction and NP stiffness [2, 21]
deformable/deformable	CGMD & MC/ *in vivo*	NP flexibility and membrane undulations dictate the receptor-ligand translational entropy making the entropy compensation context-specific [1, 22]
Semi-rigid/deformable	MC/*in vitro*	Receptor-ligand binding increases non-linearly with ligand number due to multivalent effects and membrane flexibility [22]

Table S1. Summary of model validation by comparison to experiments for binding of functionalized NPs to diffusing ICAM-1 receptors on a fluctuating cell membrane.

Free energy (ΔF)				
**Receptor density (N_r)**	**RG**	**SRG**	**LSt**	**HSt**
50	0	4.8E-3	0.38	0.32
125	0.08	4.4E-3	8.5E-6	7.8E-7

250	0.24	2.6E-6	6.9E-8	1.4E-6
500	7.4E-5	2.9E-6	2.3E-7	1.9E-8
1000	0.58	2.5E-5	5.1E-3	2.6E-12
Enthalpy (ΔH)				
**Receptor density (N_r)**	**RG**	**SRG**	**LSt**	**HSt**
50	0	9.3E-3	0.07	0.28
125	0.12	3.3E-4	0.8	0.31
250	0.25	2.4E-4	0.8	0.26
500	6.5E-03	6.5E-4	0.005	0.37
1000	0.95	2.4E-3	0.6	0.28
Entropy ($T\Delta S$)				
**Receptor density (N_r)**	**RG**	**SRG**	**LSt**	**HSt**
50	0	9.8E-3	0.85	0.3
125	0.8	0.24	0.81	0.34
250	0.43	0.05	0.27	0.06
500	0.19	0.03	0.57	0.71
1000	0.89	0.01	0.03	0.19

Table S2. Statistical evaluation (p-values) of energy components between 6% and 20% membrane excess areas across various NP types and receptor densities in the strong-binding system.

References

1. Farokhirad, S., R.P. Bradley, and R. Radhakrishnan, *Thermodynamic analysis of multivalent binding of functionalized nanoparticles to membrane surface reveals the importance of membrane entropy and nanoparticle entropy in adhesion of flexible nanoparticles.* Soft Matter, 2019. **15**(45): p. 9271-9286.
2. Farokhirad, S., N. Ramakrishnan, D.M. Eckmann, P.S. Ayyaswamy, and R. Radhakrishnan, *Nanofluid dynamics of flexible polymeric nanoparticles under wall confinement.* Journal of Heat Transfer, 2019. **141**(5): p. 052401.
3. Ramakrishnan, N., R.W. Tourdot, D.M. Eckmann, P.S. Ayyaswamy, V.R. Muzykantov, and R. Radhakrishnan, *Biophysically inspired model for functionalized nanocarrier adhesion to cell surface: roles of protein expression and mechanical factors.* Royal Society Open Science, 2016. **3**(6): p. 160260.
4. Helfrich, W., *Elastic properties of lipid bilayers: theory and possible experiments.* Zeitschrift für Naturforschung c, 1973. **28**(11-12): p. 693-703.
5. Ramakrishnan, N., P.S. Kumar, and R. Radhakrishnan, *Mesoscale computational studies of membrane bilayer remodeling by curvature-inducing proteins.* Physics reports, 2014. **543**(1): p. 1-60.
6. Ferrer, M.C.C., S. Dastgheyb, N.J. Hickok, D.M. Eckmann, and R.J. Composto, *Designing nanogel carriers for antibacterial applications.* Acta biomaterialia, 2014. **10**(5): p. 2105-2111.

7. Carme Coll Ferrer, M., P. Sobolewski, R.J. Composto, and D.M. Eckmann, *Cellular uptake and intracellular cargo release from dextran based nanogel drug carriers.* Journal of nanotechnology in engineering and medicine, 2013. **4**(1): p. 011002.
8. Coll Ferrer, M.C., R.C. Ferrier, D.M. Eckmann, and R.J. Composto, *A facile route to synthesize nanogels doped with silver nanoparticles.* Journal of nanoparticle research, 2013. **15**: p. 1-7.
9. Sarkar, A., D.M. Eckmann, P.S. Ayyaswamy, and R. Radhakrishnan, *Hydrodynamic interactions of deformable polymeric nanocarriers and the effect of crosslinking.* Soft Matter, 2015. **11**(29): p. 5955-5969.
10. Weeks, J.D., D. Chandler, and H.C. Andersen, *Role of repulsive forces in determining the equilibrium structure of simple liquids.* The Journal of chemical physics, 1971. **54**(12): p. 5237-5247.
11. Bell, G.I., *Models for the specific adhesion of cells to cells: a theoretical framework for adhesion mediated by reversible bonds between cell surface molecules.* Science, 1978. **200**(4342): p. 618-627.
12. Metropolis, N., A.W. Rosenbluth, M.N. Rosenbluth, A.H. Teller, and E. Teller, *Equation of state calculations by fast computing machines.* The journal of chemical physics, 1953. **21**(6): p. 1087-1092.
13. Ramakrishnan, N., P. Sunil Kumar, and J.H. Ipsen, *Monte Carlo simulations of fluid vesicles with in-plane orientational ordering.* Physical Review E—Statistical, Nonlinear, and Soft Matter Physics, 2010. **81**(4): p. 041922.
14. Liu, J., et al., *Computational model for nanocarrier binding to endothelium validated using in vivo, in vitro, and atomic force microscopy experiments.* Proceedings of the National Academy of Sciences, 2010. **107**(38): p. 16530-16535.
15. Agrawal, N.J. and R. Radhakrishnan, *The role of glycocalyx in nanocarrier-cell adhesion investigated using a thermodynamic model and Monte Carlo simulations.* The Journal of Physical Chemistry C, 2007. **111**(43): p. 15848-15856.
16. Frenkel, D. and B. Smit, *Understanding molecular simulation: from algorithms to applications*. 2023: Elsevier.
17. Andricioaei, I. and M. Karplus, *On the calculation of entropy from covariance matrices of the atomic fluctuations.* The Journal of Chemical Physics, 2001. **115**(14): p. 6289-6292.
18. Carlsson, J. and J. Åqvist, *Absolute and relative entropies from computer simulation with applications to ligand binding.* The Journal of Physical Chemistry B, 2005. **109**(13): p. 6448-6456.
19. Balsera, M.A., W. Wriggers, Y. Oono, and K. Schulten, *Principal component analysis and long time protein dynamics.* The Journal of Physical Chemistry, 1996. **100**(7): p. 2567-2572.
20. Glasstone, S., *Thermodynamics for chemists.* (No Title), 1947.
21. Farokhirad, S., et al., *Stiffness can mediate balance between hydrodynamic forces and avidity to impact the targeting of flexible polymeric nanoparticles in flow.* Nanoscale, 2019. **11**(14): p. 6916-6928.
22. Farokhirad, S., S.K. Kandy, A. Tsourkas, P.S. Ayyaswamy, D.M. Eckmann, and R. Radhakrishnan, *Biophysical considerations in the rational design and cellular targeting of flexible polymeric nanoparticles.* Advanced materials interfaces, 2021. **8**(23): p. 2101290.

23. McKenzie, M., S.M. Ha, A. Rammohan, R. Radhakrishnan, and N. Ramakrishnan, *Multivalent binding of a ligand-coated particle: role of shape, size, and ligand heterogeneity.* Biophysical Journal, 2018. **114**(8): p. 1830-1846.
